# The Hippo pathway mediates inhibition of vascular smooth muscle cell proliferation by cAMP

**DOI:** 10.1016/j.yjmcc.2015.11.024

**Published:** 2016-01

**Authors:** Tomomi E. Kimura, Aparna Duggirala, Madeleine C. Smith, Stephen White, Graciela B. Sala-Newby, Andrew C. Newby, Mark Bond

**Affiliations:** Bristol Heart Institute, School of Clinical Sciences, University of Bristol, Bristol BS2 8HW, UK

**Keywords:** YAP, TAZ, TEAD, cAMP, 3′-5′-Cyclic adenosine monophosphate, VSMC

## Abstract

**Aims:**

Inhibition of vascular smooth muscle cell (VSMC) proliferation by intracellular cAMP prevents excessive neointima formation and hence angioplasty restenosis and vein-graft failure. These protective effects are mediated via actin-cytoskeleton remodelling and subsequent regulation of gene expression by mechanisms that are incompletely understood. Here we investigated the role of components of the growth-regulatory Hippo pathway, specifically the transcription factor TEAD and its co-factors YAP and TAZ in VSMC.

**Methods and results:**

Elevation of cAMP using forskolin, dibutyryl-cAMP or the physiological agonists, Cicaprost or adenosine, significantly increased phosphorylation and nuclear export YAP and TAZ and inhibited TEAD-luciferase report gene activity. Similar effects were obtained by inhibiting RhoA activity with C3-transferase, its downstream kinase, ROCK, with Y27632, or actin-polymerisation with Latrunculin-B. Conversely, expression of constitutively-active RhoA reversed the inhibitory effects of forskolin on TEAD-luciferase. Forskolin significantly inhibited the mRNA expression of the pro-mitogenic genes, *CCN1*, *CTGF*, *c-MYC* and *TGFB2* and this was reversed by expression of constitutively-active YAP or TAZ phospho-mutants. Inhibition of YAP and TAZ function with RNAi or Verteporfin significantly reduced VSMC proliferation. Furthermore, the anti-mitogenic effects of forskolin were reversed by overexpression of constitutively-active YAP or TAZ.

**Conclusion:**

Taken together, these data demonstrate that cAMP-induced actin-cytoskeleton remodelling inhibits YAP/TAZ–TEAD dependent expression of pro-mitogenic genes in VSMC. This mechanism contributes novel insight into the anti-mitogenic effects of cAMP in VSMC and suggests a new target for intervention.

## Highlights

• Elevated cAMP induces phosphorylation and nuclear export of YAP and TAZ in VSMC.
• Elevated cAMP inhibits TEAD-dependent transcription of proliferation genes in VSMC.
• YAP and TAZ are essential for VSMC proliferation.
• The anti-mitogenic effects of cAMP in VSMC are dependent on inhibition of YAP/TAZ–TEAD mediated gene expression.

## Graphical abstract

Elevated cAMP inhibits the activity of Rho GTPases and thus antagonises actin polymerisation. Impaired actin polymerisation induces the phosphorylation and nuclear export of YAP and TAZ. The nuclear export of YAP and TAZ reduces the activity of TEAD transcription factors and the transcription of TEAD-dependent cell-cycle genes. This mechanism underlies the anti-mitogenic effects of cAMP in vascular smooth muscle cells.

**Figure:**
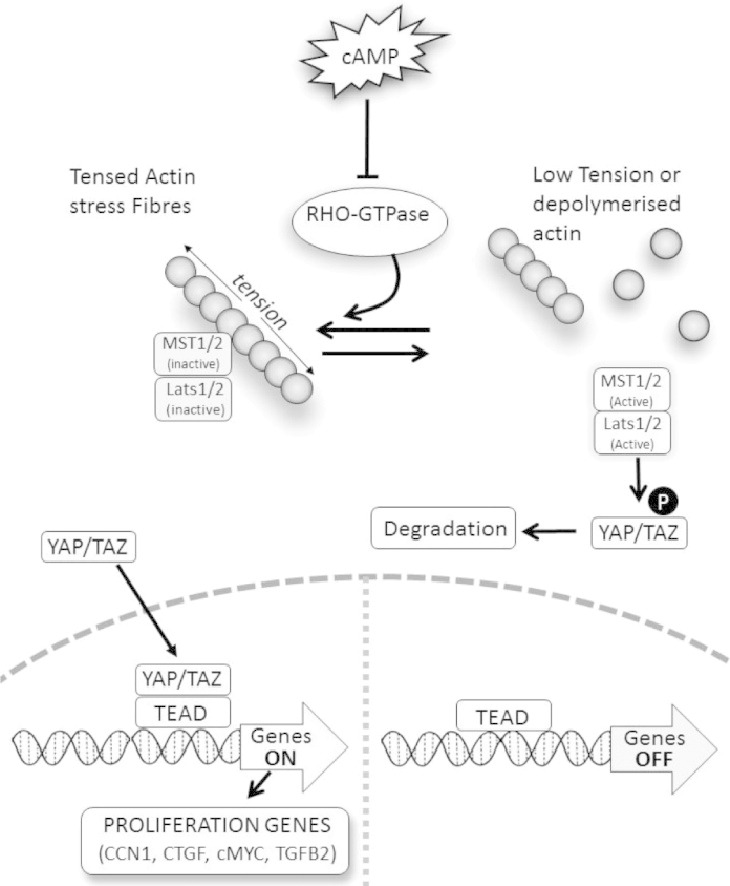

## 1 Introduction

Vascular smooth muscle cell (VSMC) proliferation contributes towards the development of various vascular diseases characterised by pathological intima formation, including atherosclerosis, transplant vasculopathy and pulmonary hypertension. Increased VSMC proliferation also contributes towards neointima formation after balloon angioplasty with and without stenting and vein grafting, limiting long-term success of clinical interventions designed to treat atherosclerosis. Except in the case of restenosis, the adverse consequences of neointima formation have remained intractable to therapy. A complete understanding of the mechanisms regulating VSMC proliferation is therefore essential for the development of new treatments.

The second messenger 3′-5′ cyclic adenosine monophosphate (cAMP) is synthesized in cells by adenylyl cyclase enzymes in response to vasoactive G_s_ protein-coupled receptor stimulation. In VSMC, elevated cAMP levels inhibit mitogen stimulated proliferation in vitro [1] and after injury in vivo [2]. Inhibitory effects of cAMP on VSMC migration [3], [4] also help reduce intimal lesion formation in experimental vascular injury models [2], [5]. As a consequence, altered or aberrant cAMP signalling is implicated in the development of many vascular pathologies [6], [7], including late vein-graft failure [8], [9], [10], angioplasty restenosis [11], atherogenesis [12] and pulmonary hypertension. For example, cAMP synthesis is impaired in response to hypertension [13], vein grafting [10], hypoxia and ox-LDL [14], suggesting that deregulation of cAMP signalling contributes towards the development of vascular pathologies.

Activation of the cAMP sensitive protein kinase A (PKA) and subsequent phosphorylation of CREB is one well-established mechanism underlying cAMP-dependent cellular responses [15]. Activation of the PKA-CREB pathway is necessary but alone insufficient for inhibition of VSMC proliferation [16], [17]. Indeed, contradictory findings suggest that CREB might also increase proliferation in some circumstances [18]. Clearly factors other than the PKA-CREB axis are involved.

Recent evidence from our laboratory and others implicates actin-cytoskeleton remodelling as an additional and essential component of cAMP signalling in VSMC [16], [19], [20], [21]. Elevated cAMP inhibits the activity of members of the Rho GTPases, which in VSMC results in remodelling of the actin-cytoskeleton and acquisition of a ‘stellate’ like morphology [19], [21]. RhoA or Rac1 inhibition using pharmacological inhibitors, siRNA or dominant–negative approaches mimics the effects of cAMP on VSMC morphology, proliferation, migration and relaxation in vitro and in vivo [19], [21]. Moreover, expression of constitutively-active RhoA or Rac1 mutants prevent cAMP-induced actin-cytoskeleton remodelling and reverse the anti-mitogenic effects of cAMP in VSMC [19], [20], [21], demonstrating the functional role of Rho GTPase inhibition. Although inhibition of Rho GTPases and actin-remodelling is an important mechanism underlying cAMP-dependent biological responses in VSMC, the downstream pathways have remained unclear.

The transcriptional co-activators Yes Associated Protein (YAP) and Transcriptional Coactivator With PDZ-Binding Motif (TAZ) have been implicated in growth regulation in numerous cell types. Overexpression of YAP homolog Yorkie in *Drosophila* induces overgrowth of fly imaginal discs [22], whilst transgenic mice overexpressing YAP develop multiple tumours [23], [24]. Likewise, several studies have linked expression of the YAP paralog TAZ to growth regulation of numerous cell types [25], [26], [27]. Although YAP and TAZ (collectively referred to as YAP/TAZ herein) can interact with several transcription factors, their growth promoting properties are primarily mediated via interaction with members of the TEAD family of transcription factors. For example, silencing of TEAD blocks expression of the majority of YAP inducible genes and largely attenuates YAP-induced overgrowth phenotype [28]. Furthermore, the phenotype of the TEAD1/2-null mice resembles the phenotype of YAP-null mice [29]. Likewise, in *Drosophila*, the TEAD-homolog mediates the overgrowth phenotype of the YAP-homolog. The transcriptional activity of the YAP/TAZ–TEAD complex is regulated by the Hippo pathway, which is centred on two kinases; MST1/2 and Lats1/2 [30]. In mammals, activation of MST1/2 kinase triggers the phosphorylation and activation of Lats 1/2 kinase, which then directly phosphorylates YAP and TAZ. Phosphorylated YAP and TAZ bind to 14-3-3ε proteins in the cytoplasm, thereby hindering their nuclear import and thus blocking their function. Importantly, in mammals the activity of MST1/2 kinase appears to be dependent on the organisation and integrity of the actin-cytoskeleton. For example, deletion of actin capping proteins or overexpression of actin nucleation factors in *Drosophila* leads to disruption of Hippo signalling [31]. In mammals, disruption of the actin-cytoskeleton induced by actin-depolymerising drugs or mechanical cues (impaired cell spreading or soft matrices) or have all been shown to induce YAP/TAZ phosphorylation [32]. In this study, we investigated if cAMP-induced remodelling of the actin-cytoskeleton regulates the activity of the YAP/TAZ–TEAD pathway and if this mechanism underlies the anti-mitogenic properties of cAMP in VSMC.

## 2 Material and methods

### 2.1 Materials

Male Sprague Dawley (SD) rats were obtained from Charles River. Culture media and additives were obtained from Invitrogen. All chemicals were obtained from Sigma unless otherwise stated. BAY60-6583 was from Tocris. Antibodies to YAP, phospho-YAPS127, phospho-YAPS397, TAZ, pan-TEAD and phospho-Retinoblastoma protein were from Cell Signalling Technologies. Anti-BrDU antibody was from Sigma.

### 2.2 Smooth muscle cell culture

Male Sprague Dawley rats were killed by cervical dislocation in accordance with the Directive 2010/63/EU of the European Parliament. Approval was granted by the University of Bristol ethical review board. Surplus segments of human aortic arch were obtained from post-mortem hearts donated for valve transplant (Research Ethical Committee number 0/H0107/48). Medial tissue was carefully dissected from the thoracic aorta and cut into 1 mm^2^ pieces for explant culture, essentially as described previously [33]. Stimulations were performed in 5% foetal calf serum/DMEM unless otherwise stated. Proliferation was measured by culture in the presence of 10 μM BrDU for 6 h. Following fixation in 70% ethanol, incorporated BrDU was detected by immune-histochemical staining as previously described [16]. Typically, all cells (at least 200) in five to ten fields of view were manually counted using ImageJ software. For nuclear and cytosolic fractionation, cells were lysed in hypotonic lysis buffer (10 mM HEPES pH 7.4, 10 mM KCl, 1.5 mM MgCl_2_, 0.05% NP-40, 1 mM PMSF, 1 μg/ml aprotinin, 1 μg/ml leupeptin) with homogenisation. Nuclei were pelleted at 1000 g and washed in lysis buffer. Nuclear proteins were extracted in Laemmli sample buffer (1% SDS, 10 mM Tris pH 6.8, 10% glycerol).

### 2.3 Quantitative RT-PCR and Western blotting

Quantification of mRNA and protein levels was performed by qRT-PCR and Western blotting respectively, essentially as described previously [16]. Total RNA, extracted using Ambion Pure-Link kits and was reverse transcribed using QuantiTect RT kit (Qiagen) and random primers. Quantitative PCR was performed using Roche SYBR Green using a BioRad Roto-Gene Q PCR machine (20′@95 °C; 20′@62 °C; 20′@72 °C). Primers sequences are described in supplement table 1. Data was normalised to total RNA levels in each reaction. Primers sequences are detailed in Table 1. Western blots were performed using a Mini-Protean II system. Proteins were transferred to PVDF membrane using a semi-dry Turbo blotter system (Bio-Rad) and detected using ECL and a digital ChemiDoc imaging system (Bio-Rad). Phos-tag gels were prepared containing 100 μM Phos-tag acrylamide and 20 μM MnCl_2_ according to the manufacturer's instructions (Alpha Laboratories).

### 2.4 Plasmids, siRNA and adenoviral vectors

TEAD reporter 8xGTIIC-luciferase was a gift from Stefano Piccolo (Addgene plasmid #34615) [32]. TnT-minP reporter was generated by digesting 8xGTIIC-luciferase with Kpn1 and BglII to remove the TEAD elements followed by blunt end re-ligation. Plasmid pCMV-YAP_S127A_ (Addgene plasmid #27370) [34] and pCMV-TAZ_4SA_ were gifts from Kunliang Guan. A 2.177 kb fragment of the human CCN1 promoter containing two proximal TEAD elements (Hg19;chr1:86044316–chr1:86046493) was described previously [35] and cloned into pGL4-luciferase (Promega). − 163 to + 57 bp fragments of the CCN1 promoter containing either wild type or mutated TEAD elements have been described previously [36]. Proximal promoter regions of the *CTGF* (Hg19;chr6:132272455–132272687) and *TGFB2* (Hg19;chr1:21851177–218520875) promoters containing consensus TEAD binding elements and TEAD ChiP-seq binding sites (ENCODE track Txn Fac ChIP V2) were amplified by PCR from human genomic DNA and cloned into the Kpn1 and Nhe1 sites of pGL4-luciferase. Recombinant adenovirus expressing constitutively-active YAP_S127A_ or TAZ_4SA_ were made as previously described by sub-cloning cDNAs from the above pCMV vectors to pDC515 (Microbix). Adenovirus expressing active RhoA_(G14V)_ has been described previously [20]. Silencer Select siRNAs targeting rat *YAP* (s170200 and s170198) and rat *TAZ* (s148961) were purchased from Life Technologies. Control siRNA was Silencer Select negative control (#4390844).

### 2.5 Transfection and recombinant adenoviruses transduction

Plasmid transfection was performed by electroporation using an Amaxa Nucleofector-1.5 machine. 1 × 10^6^ VSMC was transfected with 3–5 μg of DNA or 100 pmol of siRNA using the standard Amaxa Nucleofector program A033. VSMCs were infected with adenovirus at 1 × 10^8^ plaque forming units/ml for 16 h.

### 2.6 Reporter gene luciferase assays

Cells were transfected by electroporation with the indicated promoter reporter plasmids together with pTk-Renilla for normalisation. Cells were stimulated with the indicated agents 24 h post-transfection followed by lysis in Promega cell culture lysis buffer. Luciferase and Renilla activity was quantified using the dual reporter assay kit (Promega) according to the manufacturer's instructions using Glomax luminometer (Promega).

### 2.7 Statistical analysis

Statistical analysis was performed using ANOVA with Student × Newman × Keuls post-test or where appropriate a paired Student's t-test. * indicates p < 0.05, ** indicates p < 0.01, *** indicates p < 0.001.

## 3 Results

### 3.1 Elevated cAMP induces phosphorylation of YAP and TAZ in VSMC

**Fig. 1:**
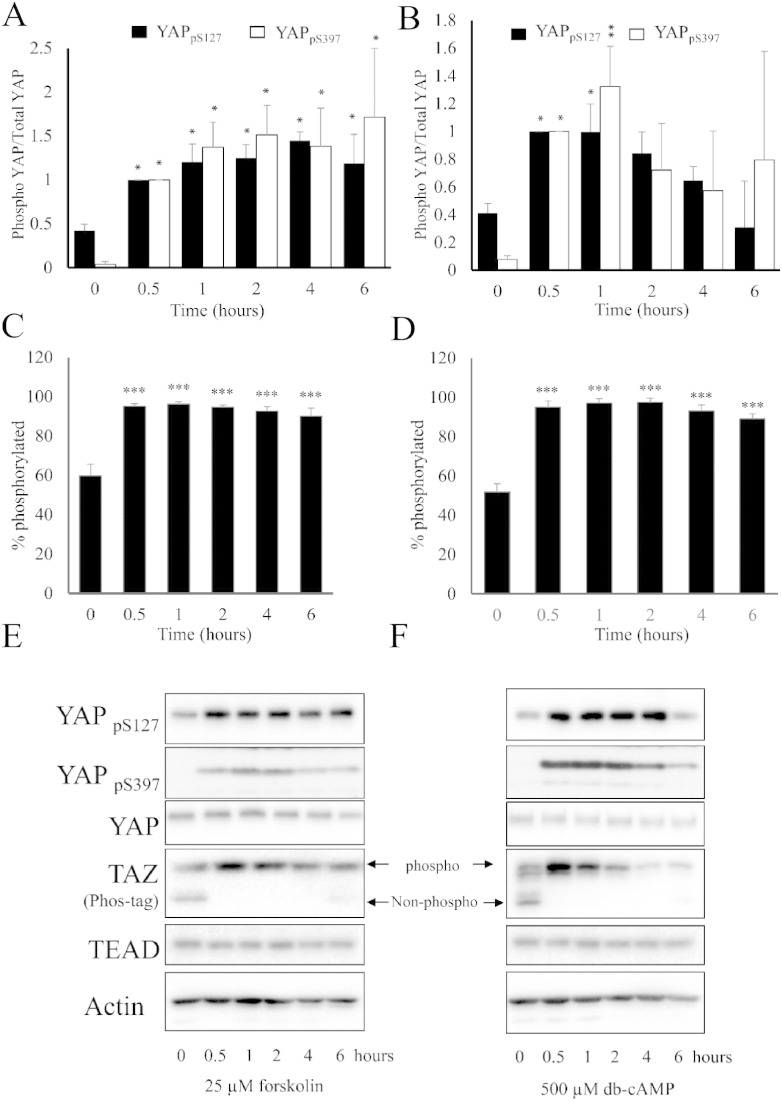
Elevated cAMP induces phosphorylation of YAP and TAZ in VSMC. VSMC were stimulated with 25 μM forskolin (A, C, E) or 500 μM db-cAMP (B, D, F) for the indicated times and total cell lysates analysed by Western blotting for phospho-YAP (S127 and S397; A, B, E, F) and total-TAZ phosphorylation (C–F). Western blots were quantified using densitometry (A–D). All experiments are at least n = 4. * indicates p < 0.05, ** indicates p < 0.01, *** indicates p < 0.001.

Elevation of intracellular cAMP induces actin-cytoskeleton remodelling and inhibits proliferation in VSMC [37], [38], [39]. We therefore hypothesised that cAMP would induce phosphorylation of downstream Hippo pathway components, YAP and TAZ, and that this would mediate growth arrest. Stimulation of cultured VSMC with the adenylate cyclase agonist, forskolin or the cAMP analogue dibutyryl-cAMP (db-cAMP) resulted in a rapid induction of a stellate morphology and loss of actin stress fibres (Supplement Fig. 1). Forskolin or db-cAMP stimulation also resulted in a rapid increase in YAP phosphorylation on serine-127 and -397 (Fig. 1A, B). Increased phosphorylation at both residues was evident after 30 min with either treatment and persisted for 6 h after forskolin stimulation and for at least 1 h after db-cAMP stimulation. Forskolin and db-cAMP stimulation increased phosphorylation of TAZ, detected by reduced electrophoretic mobility on Phos-tag acrylamide gels (Fig. 1C, D). Again, increased phosphorylation was evident after 30 min and persisted for at least 6 h. Total TEAD or β-ACTIN protein levels were not affected by forskolin stimulation. Similar increases in YAP and TAZ phosphorylation were observed after forskolin stimulation of human VSMC (Supplement Fig. 2) and in response to the physiological GPCR agonists Cicaprost, a prostacyclin mimetic, or BAY60-6583, an adenosine A2B-receptor agonist (Supplement Fig. 3).

### 3.2 Elevated cAMP induces nuclear export of YAP and TAZ, TAZ degradation and inhibition of TEAD-activity

**Fig. 2:**
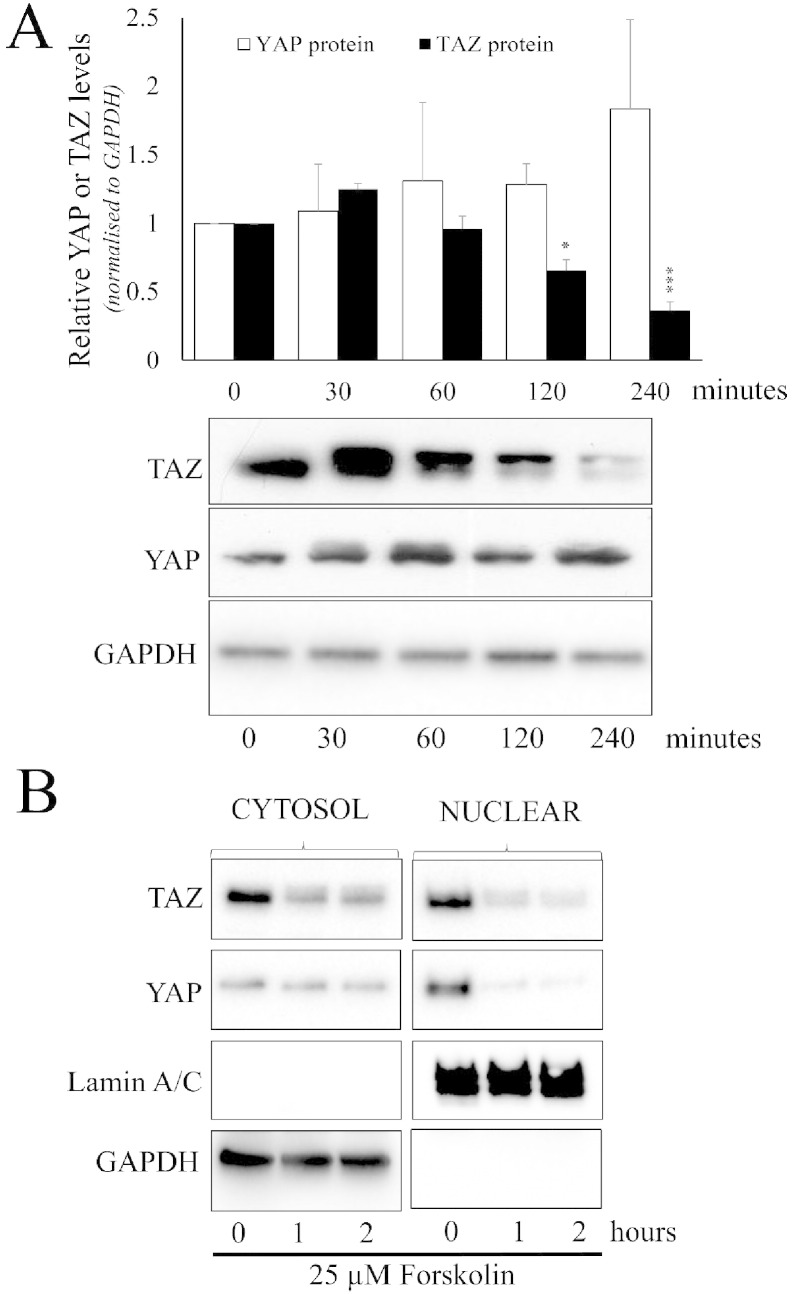
Elevated cAMP induces YAP/TAZ nuclear export and TAZ degradation. VSMC were stimulated with 25 μM forskolin for the indicated times. (A) Total cell lysates were analysed fot YAP and TAZ (n = 3). (B) Cytoplasmic and nuclear fractions were analysed for YAP, TAZ, Lamin A/C and GAPDH. *** indicates p < 0.001.

**Fig. 3:**
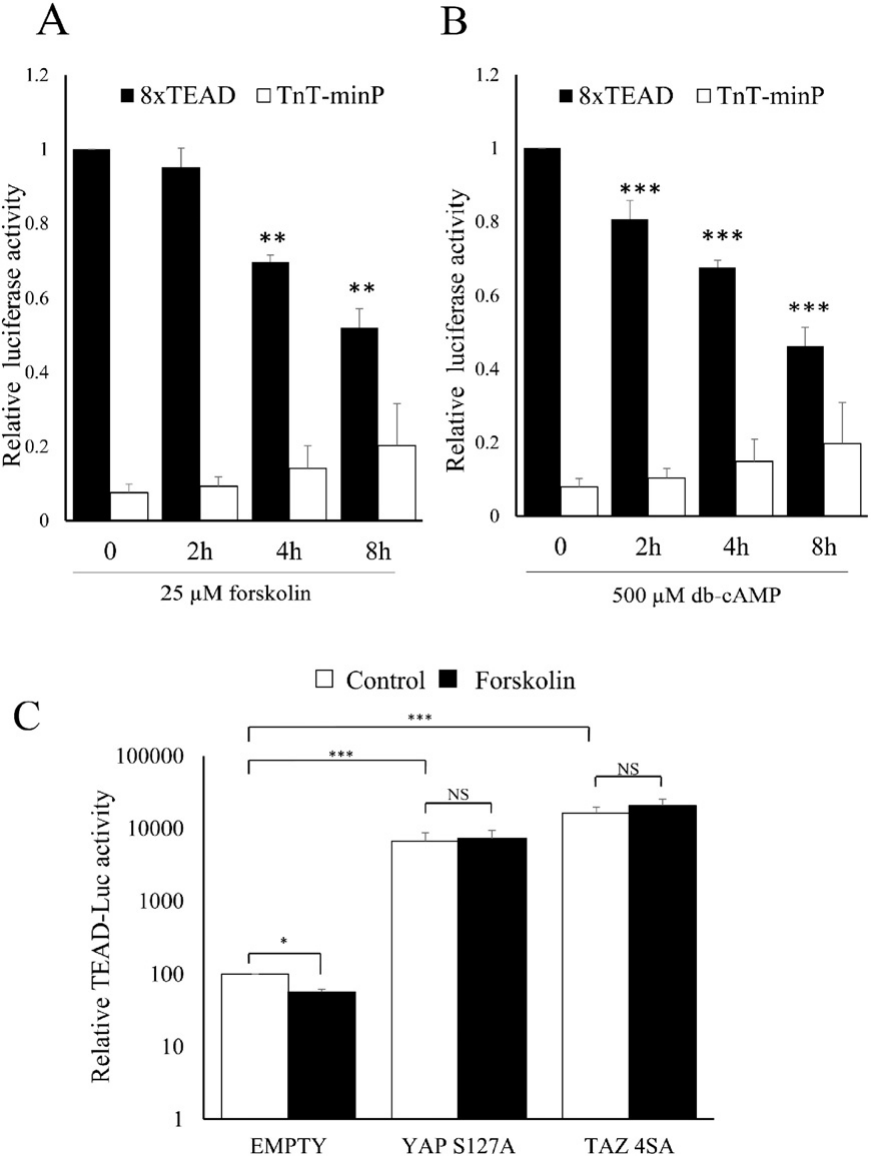
Elevated cAMP inhibited TEAD-dependent transcription. VSMCs were transfected with either TEAD-luciferase plasmid (8xTEAD) or TnT-minimal promoter-luciferase plasmid (TnT-minP) that lacks TEAD elements. Cells were stimulated with 25 μM forskolin (A and C) or 500 μM db-cAMP (B) for the indicated times. Cell lysates were assayed for luciferase activity. (C) VSMCs were transfected with TEAD-luciferase plasmid together with active YAP (YAP_S127A_), active TAZ (TAZ_4SA_), or empty control vector. The cells ere stimulated with 25 μM forskolin for 8 h. All data is n = 3 * indicates p < 0.05, ** indicates p < 0.01, *** indicates p < 0.001.

We next investigated the functional consequences of cAMP-induced YAP/TAZ phosphorylation. We initially investigated effects of elevated cAMP on total YAP and TAZ levels, since YAP phosphorylation on serine-397 has previously been a linked to YAP degradation [40]. However, forskolin stimulation of VSMC did not result in any detectable change in total YAP levels over the 240 min time course studied (Fig. 2A). In contrast, TAZ levels were significantly reduced after 120 or 240 min stimulation. We next studied the effect of forskolin on cellular localisation of YAP/TAZ using cellular fractionation. Efficient cytoplasmic and nuclear fractionation was confirmed by detection of cytoplasmic GAPDH and nuclear Lamin A/C proteins (Fig. 2B). Forskolin stimulation dramatically reduced nuclear YAP and TAZ levels after 1 and 2 h. To test whether loss of nuclear YAP/TAZ resulted in impaired TEAD activity, we quantified the activity of a synthetic TEAD-dependent luciferase reporter (8xGTIIC-luciferase) in response to cAMP-elevation. Stimulation with forskolin or db-cAMP significantly inhibited TEAD reporter activity after 4 and 8 h; inhibition was as early as 2 h with db-cAMP (Fig. 3A, B). Forskolin or db-cAMP treatment did not significantly affect the activity of a matched control reporter vector (TnT-minP) lacking the TEAD binding elements (Fig. 3A, B). Importantly, forced expression of constitutively-active phospho-site mutants of YAP (YAP_S127A_) or TAZ (TAZ_4SA_) significantly increased basal TEAD-activity and completely reversed the inhibitory effects of forskolin (Fig. 3C).

### 3.3 Inhibition of RhoA/ROCK-mediated actin polymerisation underlies cAMP-mediated repression of YAP/TAZ and TEAD

**Fig. 4:**
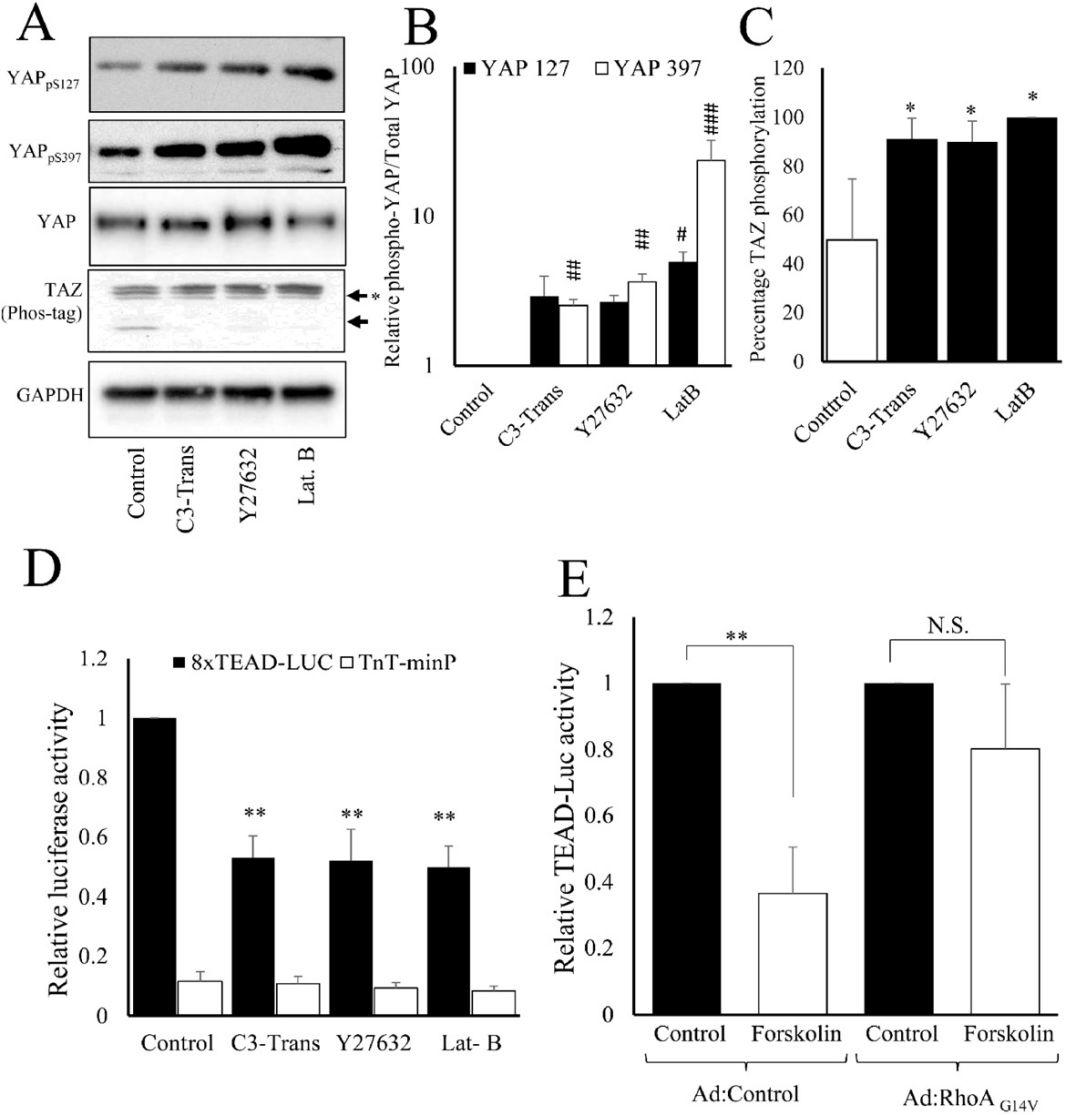
Inhibition of RhoA/ROCK-mediated actin polymerisation underlies cAMP-mediated repression of YAP/TAZ and TEAD. VSMCs were treated with RhoA inhibitor (C3 Transferase; 2 μg/ml), ROCK inhibitor (Y-27,632; 10 μM) or actin-polymerisation inhibitor (Latrunculin B; 5 μg/ml) for 30 min and phosphorylation of YAP/TAZ was quantified Western blotting (A–C). VSMCs were transfected with TEAD-luciferase plasmid (8xTEAD-LUC) or TnT-minimal promoter-luciferase plasmid (TnT-minP) and treated with indicated RhoA/ROCK pathway inhibitors for 8 h (D). Cells infected with either active RhoA (RhoA_G14V_) or control adenovirus were stimulated with 25 μM forskolin for 8 h (E). All data is n = 3. * indicates p < 0.05, ** indicates p < 0.01. ## indicates p < 0.01 and ### indicates p < 0.001 on log transformed data.

Elevated cAMP is known to inhibit RhoA activity and actin-polymerisation [20], [21], which is involved in cAMP-induced morphological changes and actin-cytoskeleton remodelling. We therefore asked whether RhoA, its effector ROCK and actin-cytoskeleton organisation are responsible for the effects of cAMP on YAP/TAZ phosphorylation and inhibition of TEAD reporter activity. Incubation with the RhoA inhibitor, C3 transferase, the ROCK inhibitor, Y27632 or the actin-depolymerisation agent, latrunculin-B, significantly increased phosphorylation of YAP and TAZ (Fig. 4A, B and C) and significantly inhibited activity of TEAD-luciferase (Fig. 4D) without affecting activity of the control reporter (TnT-minP), consistent with impaired YAP/TAZ function. Importantly, forced expression of constitutively-active RhoA, which prevents cAMP-induced actin remodelling, significantly reversed the effects of forskolin on TEAD-luciferase activity (Fig. 4E).

### 3.4 Intracellular cAMP inhibits the expression YAP/TAZ-dependent pro-mitogenic genes

**Fig. 5:**
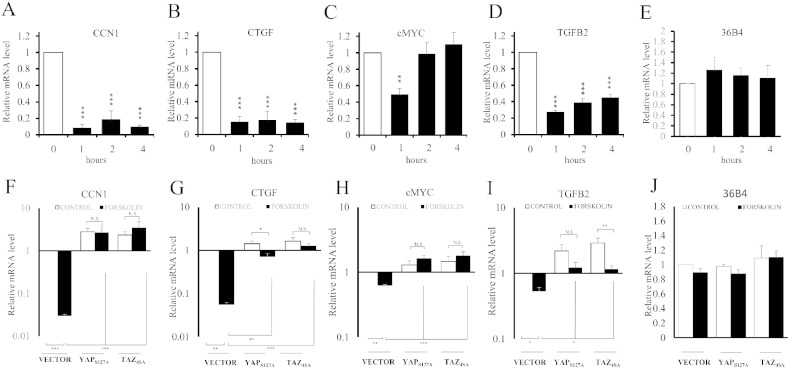
cAMP inhibits the expression YAP/TAZ-dependent pro-mitogenic genes. VSMCs were stimulated with 25 μM forskolin for the indicated times (A–F) and analysed by qRT-PCR for *CCN1* (A; n = 4), *CTGF* (B; n = 4), *cMYC* (C; n = 8), *TGFB2* (D; n = 8), *36B4* (E; n = 4). Cells were infected with control adenovirus, YAP_S127A_ adenovirus or TAZ_4SA_ adenovirus and stimulated with 25 μM forskolin (F–J). Expression of *CCN1* (F; n = 4), *CTGF* (G; n = 4), *cMYC* (H; n = 4), *TGFB2* (I; n = 4), 36B4 (J; n = 4) was analysed by qRT-PCR. * indicates p < 0.05, ** indicates p < 0.01, *** indicates p < 0.001.

Since elevated cAMP is known to potently inhibit cell-cycle progression in VSMC [1], [41] we sought to identify YAP/TAZ-dependent genes that promote cell proliferation and are repressed by elevated cAMP. We initially identified all genes containing TEAD ChIP-seq peaks (ENCODE track Txn Fac ChIP V2) within their core (− 1000 bp to + 100 bp) promoters. This list was subsequently filtered to identify genes with the gene ontology tag GO:0008284 (positive regulation of proliferation), resulting in putative YAP/TAZ–TEAD-dependent genes that are involved in the positive regulation of cell proliferation. This list contained the genes *CCN1*, *CTGF*, *c-MYC* and *TGFβ2*. The YAP/TAZ-dependence of these genes in VSMC was initially confirmed using siRNA-mediated silencing of *YAP*/*TAZ*. Transient transfection with siRNA targeting YAP or TAZ significantly repressed *YAP* or *TAZ* mRNA levels (supplement fig. 4), respectively, without affecting the mRNA levels of the housekeeping genes *GAPDH* (Supplement Fig. 4) or *36B4* (Supplement Fig. 5) or the non-TEAD target gene *EGR1* (Supplement Fig. 5). *YAP* and *TAZ* double knock-down significantly repressed mRNA levels of *CCN1*, *CTGF*, *c-MYC* and *TGFB2*, thus confirming the YAP/TAZ-dependence of these genes in VSMC (Supplement Fig. 5). We therefore tested the hypothesis that the expression of these genes would be repressed by elevated cAMP in VSMC. Forskolin stimulation significantly inhibited the mRNA levels of *CCN1*, *CTGF*, and *TGFB2* after 1, 2 and 4 h stimulation (Fig. 5A–D). Expression of c-MYC was also significantly inhibited but only after 1 h. Levels of *36B4* mRNA were unaffected by forskolin stimulation (Fig. 5E). To test whether this inhibition was dependent on YAP and/or TAZ inactivation, we investigated the effect of constitutively-active mutants of YAP and TAZ. Forced expression of constitutively-active YAP (YAP_S127A_) or TAZ (TAZ_4SA_) significantly increased basal expression of all four genes (Fig. 5F–I), consistent with YAP/TAZ–TEAD-dependent expression of these genes. More importantly, the forskolin-mediated repression of these mRNAs was significantly reversed by YAP_S127A_ or TAZ_4SA_ (Fig. 5F–I). Levels of *36B4* mRNA were unaffected by YAP_S127A_ or TAZ_4SA_ expression (Fig. 5J). To investigate effects on the promoter activity of these genes, we cloned their promoter regions encompassing the proximal TEAD-binding elements, upstream of luciferase. Forskolin-mediated inhibition of *CCN1*-LUC, *CTGF*-LUC and *TGFB2*-LUC promoter activity was similarly reversed by forced expression of YAP_S127A_ or TAZ_4SA_ (Supplement Fig. 6). Taken together, these data demonstrate that elevated cAMP represses expression of pro-mitogenic genes in VSMC by inhibiting the activity of YAP/TAZ. Furthermore, mutation of TEAD-binding element 1 in the CCN1 promoter significantly inhibited CCN1 promoter activity (Supplement Fig. 7), consistent with a functional role for TEAD in CCN1 expression in VSMC.

### 3.5 Inhibition of YAP and TAZ underlies cAMP anti-mitogenesis in VSMC

**Fig. 6:**
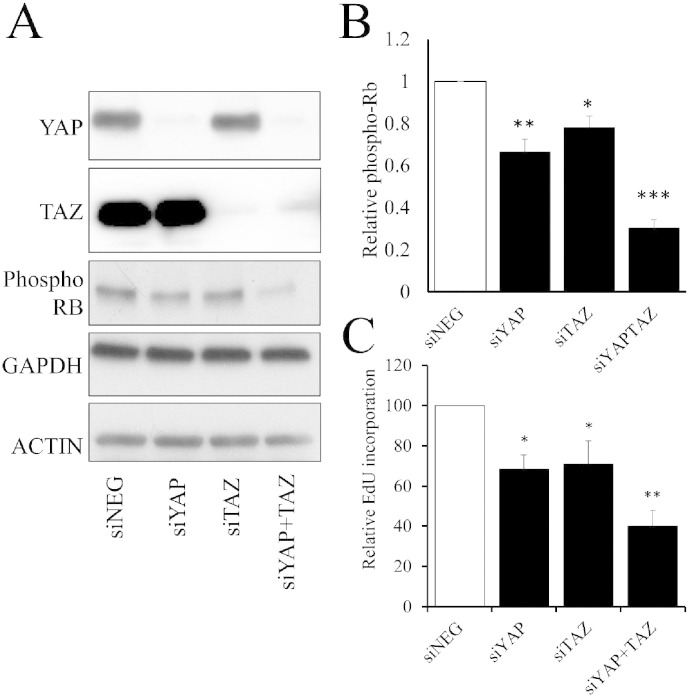
YAP and TAZ cooperate to regulate VSMC proliferation. VSMC were transfected with siRNA targeting YAP, TAZ or YAP plus TAZ. Total cell lysates were prepared 24 h post transfection and analysed by Western blotting for YAP, TAZ, phospho-Rb, GAPDH and ACTIN (A). Phospho-Rb levels were quantified by densitometry (B; n = 3). siRNA-transfected cells were labelled with BrdU between 24 and 36 h post transfection (C; n = 3). * indicates p < 0.05, ** indicates p < 0.01, *** indicates p < 0.001.

We next tested the functional importance of YAP/TAZ for VSMC proliferation and cAMP-mediated growth arrest. VSMC were transiently transfected with siRNA targeting YAP, TAZ or both YAP and TAZ together. YAP siRNA and TAZ siRNA significantly inhibited the mRNA (Supplement Fig. 4) and protein levels (Fig. 6A) of YAP and TAZ respectively, whilst dual YAP/TAZ siRNA significantly repressed both proteins. Importantly, expression of GAPDH or ACTIN protein remained unaffected. Silencing of YAP or TAZ resulted in a significant reduction in the levels of hyper-phosphorylated retinoblastoma protein (Rb), a marker of transition through the G_1_-S phase checkpoint. Dual silencing of YAP/TAZ had an additive effect, further reducing phospho-Rb levels (Fig. 6A and B). Silencing of YAP or TAZ also resulted in a significant inhibition of VSMC proliferation (quantified by BrDU incorporation), whilst dual silencing of YAP/TAZ again had an additive effect (Fig. 6C and Supplement Fig. 8). Taken together, these data demonstrate that both YAP and TAZ are important mediators of VSMC proliferation.

**Fig. 7:**
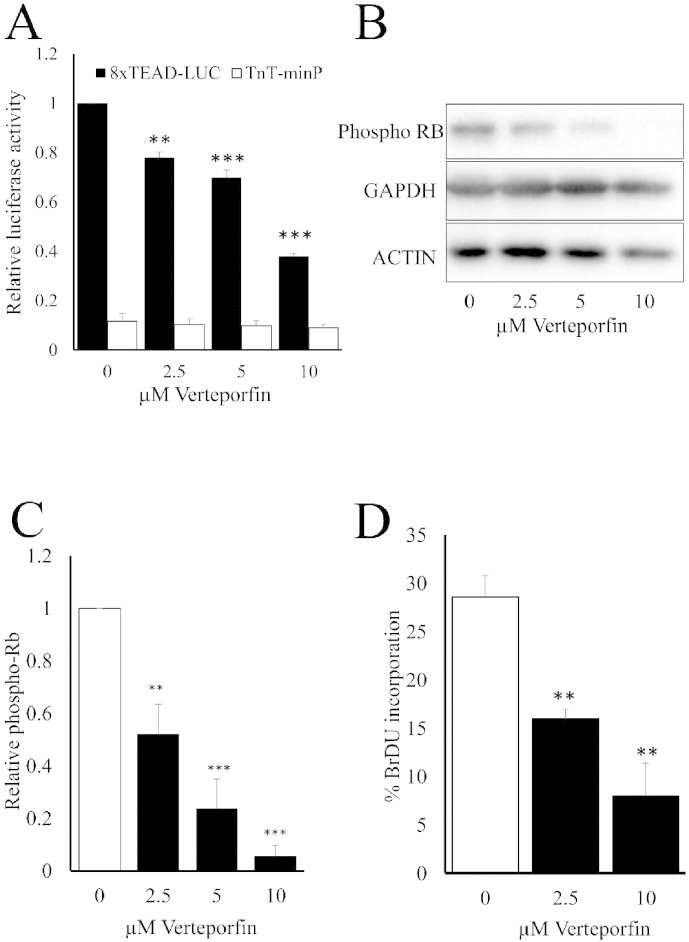
The YAP–TEAD inhibitor Veteporfin inhibits VSMC proliferation. VSMC were transfected with TEAD-luciferase reporter plasmid (8xTEAD) or a TnT-minimal promoter-luciferase plasmid (TnT-minP) and treated with the indicated concentrations of Verteporfin for 8 h (A; n = 3). VSMCs were treated with the indicated concentrations of Verteporfin for 18 h and total cell lysates analysed for phospho-Rb, GAPDH and ACTIN levels by Western blotting (B). Phospho-Rb Western blots were quantified by densitometry (C; n = 3). VSMCs were treated with Verteporfin for 18 h followed by BrdU labelling for 6 h (D; n = 3). * indicates p < 0.05, ** indicates p < 0.01, *** indicates p < 0.001.

To further confirm the functional importance of YAP/TAZ in VSMC proliferation we treated cells with the benzoporphyrin derivative, Verteporfin (VP), which has been shown to disrupt the interaction between YAP and TEAD [42]. We initially confirmed this in VSMC using YAP–TEAD pull-down assays (Supplement Fig. 9). VSMCs were transfected with GFP-YAP followed by treatment with VP for 2 h. GFP-YAP was affinity purified using GFP-Trap beads and co-purified TEAD quantified by Western blotting. These assays confirmed that VP inhibits the interaction between YAP and TEAD in VSMC. VP treatment also significantly inhibited TEAD-luciferase activity in a dose-dependent manner (Fig. 7A), consistent with disruption of the YAP–TEAD complex. Importantly, VP treatment significantly inhibited levels of hyper-phosphorylated-Rb protein (Fig. 7B and C) and incorporation of BrDU (Fig. 7D and Supplement Fig. 10), further confirming the involvement of YAP/TAZ–TEAD in G_1_-S phase progression and proliferation in VSMC.

**Fig. 8:**
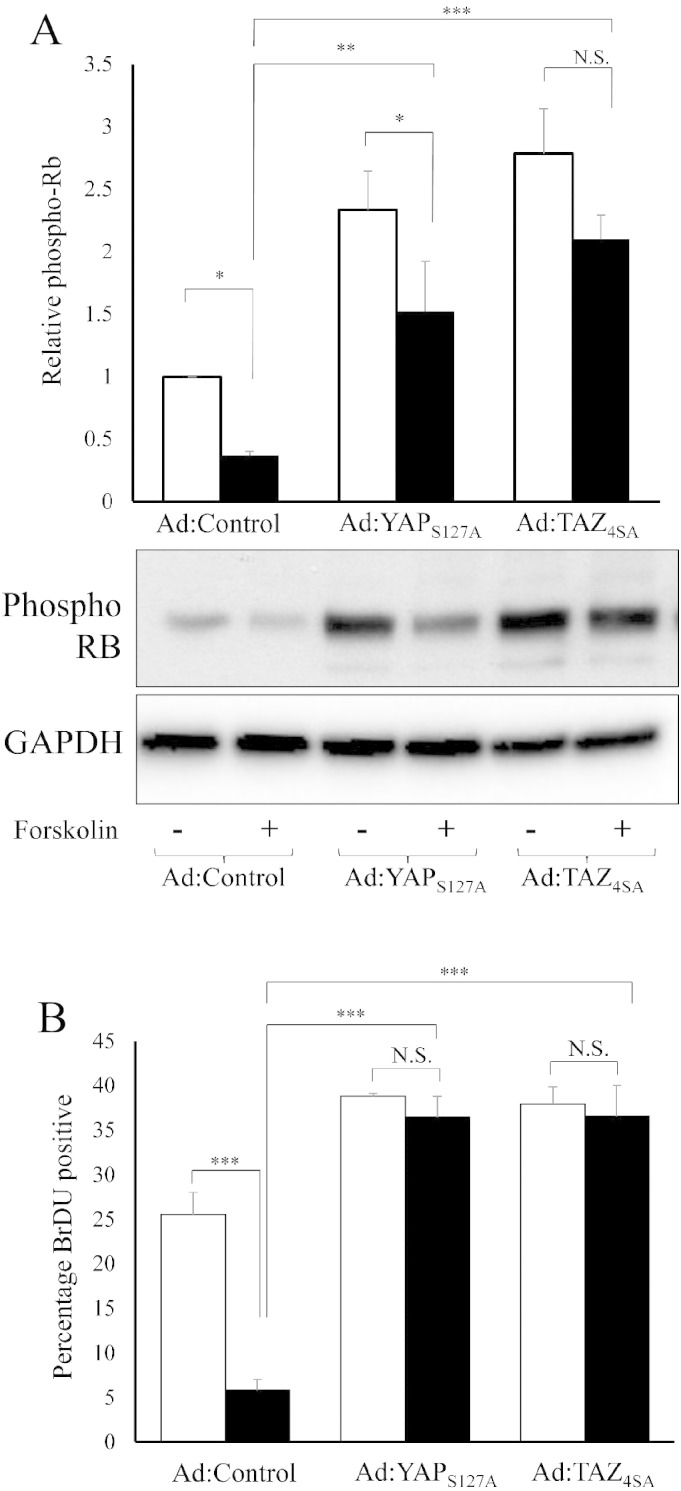
Constitutively active YAP and TAZ reverse the anti-mitogenic effect of forskolin in VSMC. VSMCs were infected with 3 × 10^7^ pfu/ml of control adenovirus or adenovirus expressing constitutively active YAP_S127A_ or TAZ_4SA_. Cells were stimulated with 25 μM forskolin for 18 h (A and B). Total cell lysates were analysed for phospho-Rb levels by Western blotting and densitometry (A; n = 3). Cells were labelled with BrDU for 6 h immediately after forskolin stimulations (B; n = 3). * indicates p < 0.05, ** indicates p < 0.01, *** indicates p < 0.001.

Lastly, we sought to test the functional importance of YAP and TAZ for the anti-mitogenic effects of cAMP in VSMC using adenovirus-mediated expression of constitutively-active YAP (YAP_S127A_) or TAZ (TAZ_4SA_). Forskolin-treatment significantly reduced levels of hyper-phosphorylated-Rb in control adenovirus infected cells (Fig. 8A), consistent with previous studies [43], [44]. Forced expression of active-YAP significantly elevated basal levels of hyper-phosphorylated-Rb (p < 0.01). Forskolin-stimulation of YAP_S127A_ expressing cells resulted in a significant inhibition of hyper-phosphorylated-Rb (p < 0.05). However, this was to levels significantly higher than in forskolin stimulated control virus infected cells (p < 0.01) and to levels that were not different from unstimulated control virus infected cells. Overexpression of TAZ_4SA_ also significantly elevated basal hyper-phosphorylated-Rb levels. Importantly, forskolin stimulation of TAZ_4SA_ expressing cells did not significantly alter hyper-phosphorylated-Rb levels (Fig. 8A). Likewise, forskolin stimulation of cells doubly infected with both YAP_S127A_ and TAZ_4SA_ expressing adenoviruses did not result in a significant inhibition of hyper-phosphorylated-Rb (Supplement Fig. 11). Forskolin stimulation of control adenovirus infected cells significantly inhibited BrDU incorporation (Fig. 8B and Supplement Fig. 12). Expression of YAP_S127A_ or TAZ_4SA_ both significantly increased basal levels of BrDU incorporation (Fig. 8B) and completely prevented the forskolin-mediated inhibition or BrDU incorporation (Fig. 8B). Taken together, these data demonstrate a role for YAP and TAZ inactivation in cAMP-mediated growth arrest.

## 4 Discussion

In this study, we investigated the role of Hippo pathway effector proteins YAP, TAZ and TEAD in cAMP-mediated growth arrest in VSMC. It was previously shown that YAP levels are elevated in both mouse and rat arterial injury models, where expression correlates with a synthetic VSMC phenotype [45]. We now show, using loss and gain of function strategies, that both YAP and TAZ are essential for proliferation in adult VSMC. We also demonstrate that cAMP-induced actin-remodelling suppresses the activity of both YAP and TAZ and that this mechanism underlies the anti-mitogenic effects of cAMP in VSMC. In detail, elevation of cAMP using the forskolin, db-cAMP or the GPCR agonists Cicaprost or BAY60-6583 all rapidly induced YAP phosphorylation (serine-127 and serine-397) and pan-TAZ phosphorylation. This rapidly reduced nuclear YAP and TAZ levels, most likely via impaired nuclear import or enhanced export. Consistent with the function of YAP and TAZ as co-factors for TEAD-family transcription factors, reduction in nuclear YAP and TAZ resulted in reduced TEAD-dependent reporter gene activity. We did not observe any effects of cAMP on total YAP levels, despite previous work implicating YAP phosphorylation on serine-397 (annotated as S-381 by Zhao [40]) in priming YAP degradation. Furthermore, we went on to demonstrate that cAMP induced growth-arrest is associated with repression of a set of YAP/TAZ-dependent proliferation genes (i.e. *CCN1*, *CTGF*, *c-MYC* and *TGFB2*) in VSMC. Expression of these genes is repressed either by YAP/TAZ silencing or cAMP elevation. Moreover, constitutively-active YAP or TAZ phospho-mutants reversed, to varying degrees, the inhibitory effects of forskolin, confirming a functional role of YAP/TAZ in mediating the effects of cAMP on these genes. Importantly, expression of all of these genes have been associated with vascular remodelling in vivo and functionally linked to increased VSMC proliferation [35], [46], [47]. Our new data suggests that expression of these genes in VSMC is dependent on YAP and TAZ and subject to repression by elevated cAMP. The implication is that inhibition of these genes contributes towards the anti-mitogenic effects of cAMP in VSMC. Our data showing increased phosphorylation of YAP and TAZ by the prostacyclin analogue also suggests that endothelial derived prostacyclin may play a role in maintaining VSMC quiescence in healthy vessels via this mechanism. Taken together, these observations indicate that cAMP-mediated inhibition of YAP and TAZ and the subsequent inhibition of TEAD-dependent transcription is an important early mechanism underlying cAMP anti-mitogenesis in VSMC.

YAP and TAZ have been previously implicated in the regulation of cell proliferation in several nonvascular cell types. For example, over expression of Yorkie, the *Drosophila* homolog of YAP results in robust tissue over growth phenotype. In mammals, forced expression of active mutants of YAP or TAZ stimulate fibroblast, epithelial and mammary carcinoma cell proliferation and migration, whereas expression of dominant-negative mutants or siRNA silencing inhibits it [48], [49], [50], [51], consistent with our findings. Complementing its role in adult VSMC, YAP also influences cardiovascular development and homeostasis. Loss of YAP in the murine cardiovascular system results in perinatal lethality, while YAP conditional knockout mice exhibit impaired embryonic VSMC proliferation leading to severe vascular abnormalities including thinning vascular walls and short/absent brachiocephalic arteries [52]. This may reflect the known role of YAP in VSMC phenotypic modulation [53]. TEAD-dependent transcription has also been linked to regulation of cell proliferation in several species and cell types. In *Drosophila*, the TEAD-homolog, Scalloped has been shown to mediate the overgrowth phenotype of the Yorkie. Furthermore, the mitogenic properties of YAP are dependent on its ability to enhance TEAD transcriptional activity, since TEAD silencing blocks expression of the majority of YAP inducible genes and largely attenuates YAP-induced overgrowth phenotype [28]. Furthermore, the phenotype of the TEAD1/2-null mice resembles the phenotype of YAP-null mice [29].

Elevated levels of cAMP arrest VSMC in G_1_ phase of the cell-cycle, at least in part through inhibition of G_1_-regulatory proteins CYCLIN-D1 and SKP2 [41], [54]. However, these events occur relatively late in G_1_-phase and these genes are unlikely to be direct targets of the YAP/TAZ–TEAD pathway. Neither contain TEAD binding elements in their proximal promoters and their repression by cAMP is delayed (taking several hours) compared to the YAP/TAZ targets we describe here. However, CYCLIN-D1 expression is enhanced in response to CTGF and CCN1 stimulation [46], [55], suggesting that CYCLIN-D1 repression may occur, at least in part, due to the early and direct effects on CCN1 and CTGF. Reduced CYCLIN-D1 expression will slow cell-cycle progression by limiting cdk4 activity and preventing E2F activation. E2F positively regulates SKP2 transcription as part of a positive auto-induction loop the drives cells into S-phase. Taken together, this suggest that cAMP-mediated inhibition of YAP/TAZ target genes not only precedes but may also contribute towards the later inhibition of CYCLIN-D1 and SKP2 and represent the earliest mechanisms underlying cAMP anti-mitogenesis. Defining these early mechanisms could have an important impact on development of new pharmacological approaches to combat cardiovascular disease.

Given the importance of YAP/TAZ in the regulation of cell proliferation, recent attention has focussed on characterising the upstream signals that regulate YAP/TAZ activity. Notably, signals that modulate actin-cytoskeleton organisation appear to be particularly important. These include mechanical, cell–cell contact and mitogenic growth factor signals. Our new data demonstrates that cAMP-mediated actin-cytoskeleton remodelling is also an important mechanism that negatively regulates YAP/TAZ activity in VSMC. Elevated cAMP inhibits the activity of members of the Rho GTPases, including RhoA and Rac1 [19], [20], [21], rapidly inhibiting actin-polymerisation and inducing a ‘stellate’ morphology, characterised by a condensed cytoplasm and loss of actin stress-fibres. Rho GTPase activity promotes VSMC proliferation and subsequent intima formation following balloon injury and forced expression of constitutively-active Rho GTPase mutants prevents cAMP-induced actin-cytoskeleton remodelling and rescues VSMC proliferation [19], demonstrating that inhibition of Rho GTPase-dependent actin-cytoskeleton polymerisation plays a decisive role in cAMP anti-mitogenesis in VSMC. Precisely how these cAMP induced actin changes modulate VSMC behaviour has remained poorly understood. Our new data suggests that cAMP counteracts the effects of diverse mitogenic signals by inhibiting Rho GTPase mediated actin-cytoskeleton remodelling. In turn, this inhibits the YAP/TAZ-TEAD-dependent expression of proliferation genes. Given that many mitogenic signals act, at least in part, via positive regulation of the RhoA-actin pathway, this implies that regulation of YAP/TAZ is an important central node for multiple mitogenic and anti-mitogenic signalling pathways and as such may represent a valuable target for future anti-proliferative therapies.

Proliferation of VSMC is a major factor that contributes towards pathological intimal thickening underlying numerous vascular pathologies, including late vein graft failure, in-stent restenosis, pulmonary artery hypertension and atherosclerosis. Intracellular cAMP is a major second messenger involved in the regulation of VSMC proliferation. The ability of vascular cells to synthesise cAMP is high in healthy vessels where it contributes towards the maintenance of VSMC quiescence. Reduced synthesis of cAMP after vessel injury or during disease is likely to be an important mechanism in removing the “brake” on proliferation. A large body of evidence documents the anti-mitogenic properties of cAMP in VSMC and ultimately its ability to repress intima formation in vivo [41]. However, translating knowledge of cAMP signalling into clinically useful therapy has also been challenging mainly owing to the multiple cell-type specific effects of the cAMP system. Direct targeting of specific cAMP-sensitive pathways may therefore be a valuable strategy to harness the beneficial effects of cAMP whilst avoiding unwanted side effects. Interestingly, the ability of the benzoporphyrin derivative, Verteporfin to block the interaction between YAP and TEAD demonstrates that this protein:protein interaction is amenable to small-molecule antagonism, raising the hope for future YAP/TAZ based therapies.

## Glossary

BrdU: 5-Bromo-2-deoxyuridine
cAMP: 3′-5′-cyclic adenosine monophosphate
CREB: cAMP Response Element Binding Protein
SRF: Serum Response Factor
VSMC: Vascular Smooth Muscle Cell
RhoA: Ras Homolog Family Member A
Rac: Ras-related C3 botulinum toxin substrate 1
VSMC: Vascular Smooth Muscle Cell
YAP: Yes-Associated Protein-1
TAZ: Transcriptional Co-Activator with PDZ-Binding Motif
TEAD: TEA Domain Family Member

## Disclosures

Conflict of interest: None.

## Figures and Tables

**Table 1:** Primer sequences used in qPCR reactions.

Primer description	Sequence (5′-3′)
Rat CCN1 forward	GGAACTGGCATCTCCACACGAGTT
Rat CCN1 reverse	TTGTCCACAAGGACGCACTTCACA
Rat CTGF forward	GGAAATGCTGTGAGGAGTGGGTGT
Rat CTGFreverse	TGTCTTCCAGTCGGTAGGCAGCTA
Rat cMYC forward	GAGGAGAAACGAGCTGAAGCGTAG
Rat cMYC reverse	TTCTCGCCGTTTCCTCAGTAAGTC
Rat TGFB2 forward	TCCATACAGTCCCAGGTGCTCTGT
Rat TGFB2 reverse	GACCCTGAACTCTGCCTTCACCAG

## References

[1] Southgate K.M., Newby A.C. (1990). Serum-induced proliferation of rabbit aortic smooth muscle cells from the contractile state is inhibited by 8-Br-cAMP but not 8-Br-cGMP. Atherosclerosis.

[2] Wu Y.J., Bond M., Sala-Newby G.B., Newby A.C. (2006). Altered S-phase kinase-associated protein-2 levels are a major mediator of cyclic nucleotide-induced inhibition of vascular smooth muscle cell proliferation. Circ. Res..

[3] Palmer D., Maurice D.H. (1997). cAMP-mediated inhibition of vascular smooth muscle cell migration: role of cAMP-phosphodiesterases. Mol. Biol. Cell.

[4] Howe A. (1692). Regulation of actin-based cell migration by cAMP/PKA. Biochim. Biophys. Acta Mol. Cell Res..

[5] Indolfi C., Di Lorenzo E., Rapacciuolo A., Stingone A.M., Stabile E., Leccia A. (2000). 8-Chloro-cAMP inhibits smooth muscle cell proliferation in vitro and neointima formation induced by balloon injury in vivo. J. Am. Coll. Cardiol..

[6] Savai R., Pullamsetti S.S., Banat G.A., Weissmann N., Ghofrani H.A., Grimminger F. (2010). Targeting cancer with phosphodiesterase inhibitors. Expert Opin. Investig. Drugs.

[7] Netherton S.J., Jimmo S.L., Palmer D., Tilley D.G., Dunkerley H.A., Raymond D.R. (2002). Altered phosphodiesterase 3-mediated cAMP hydrolysis contributes to a hypermotile phenotype in obese JCR: LA-cp rat aortic vascular smooth muscle cells - implications for diabetes-associated cardiovascular disease. Diabetes.

[8] Jeremy J.Y., Dashwood M.R., Timm M., Izzat M.B., Mehta D., Bryan A.J. (1997). Nitric oxide synthase and adenylyl and guanylyl cyclase activity in porcine interposition vein grafts. Ann. Thorac. Surg..

[9] Jeremy J.Y., Birkett S.D., Bryan A.J., Angelini G.D. (1997). The influence of surgical preparation on cyclic nucleotide synthesis in an organ culture of human saphenous vein. Eur. J. Vasc. Endovasc. Surg..

[10] Jeremy J.Y., Dashwood M.R., Mehta D., Izzat M.B., Shukla N., Angelini G.D. (1998). Nitric oxide, prostacyclin and cyclic nucleotide formation in externally stented porcine vein grafts. Atherosclerosis.

[11] Yokoyama U., Minamisawa S., Quan H., Akaike T., Jin M.H., Otsu K. (2008). Epac1 is upregulated during neointima formation and promotes vascular smooth muscle cell migration. Am. J. Physiol. Heart Circ. Physiol..

[12] Schauer I.E., Knaub L.A., Lloyd M., Watson P.A., Gliwa C., Lewis K.E. (2010). CREB downregulation in vascular disease a common response to cardiovascular risk. Arterioscler. Thromb. Vasc. Biol..

[13] Murray F., Patel H.H., Suda R.Y.S., Zhang S., Thistlethwaite P.A., Yuan J.X.J. (2007). Expression and activity of cAMP phosphodiesterase isoforms in pulmonary artery smooth muscle cells from patients with pulmonary hypertension: role for PDE1. Am. J. Physiol. Lung Cell. Mol. Physiol..

[14] Jing Q., Xin S.M., Cheng Z.J., Zhang W.B., Zhang R., Qin Y.W. (1999). Activation of p38 mitogen-activated protein kinase by oxidized LDL in vascular smooth muscle cells — mediation via pertussis toxin-sensitive G proteins and association with oxidized LDL-induced cytotoxicity. Circ. Res..

[15] Mayr B., Montminy M. (2001). Transcriptional regulation by the phosphorylation-dependent factor CREB. Nat. Rev. Mol. Cell Biol..

[16] Hewer R.C., Sala-Newby G.B., Wu Y.J., Newby A.C., Bond M. (2011). PKA and Epac synergistically inhibit smooth muscle cell proliferation. J. Mol. Cell. Cardiol..

[17] Klemm D.J., Watson P.A., Frid M.G., Dempsey E.C., Schaack J., Colton L.A. (2001). cAMP response element-binding protein content is a molecular determinant of smooth muscle cell proliferation and migration. J. Biol. Chem..

[18] Tokunou T., Ichiki T., Takeda K., Funakoshi Y., Iino N., Takeshita A. (2001). cAMP response element-binding protein mediates thrombin-induced proliferation of vascular smooth muscle cells. Arterioscler. Thromb. Vasc. Biol..

[19] Bond M., Wu Y.J., Sala-Newby G.B., Newby A.C. (2008). Rho GTPase, Rac(1), regulates Skp(2) levels, vascular smooth muscle cell proliferation, and intima formation in vitro and in vivo. Cardiovasc. Res..

[20] Kimura T.E., Duggirala A., Hindmarch C.C.T., Hewer R.C., Cui M.Z., Newby A.C. (2014). Protein kinase A and EPAC synergistically inhibit Egr1 expression and proliferation in vascular smooth muscle cells. JMCC.

[21] Pelletier S., Julien C., Popoff M., Lamarche-Vane N., Meloche S. (2005). Cyclic AMP induces morphological changes of vascular smooth muscle cells by inhibiting a Rac-dependent signaling pathway. J. Cell. Physiol..

[22] Huang J.B., Wu S., Barrera J., Matthews K., Pan D.J. (2005). The Hippo signaling pathway coordinately regulates cell proliferation and apoptosis by inactivating Yorkie, the Drosophila homolog of YAP. Cell.

[23] Camargo F.D., Gokhale S., Johnnidis J.B., Fu D., Bell G.W., Jaenisch R. (2007). YAP1 increases organ size and expands undifferentiated progenitor cells. Curr. Biol..

[24] Dong J., Feldmann G., Huang J., Wu S., Zhang N., Comerford S.A. (2007). Elucidation of a universal size-control mechanism in Drosophila and mammals. Cell.

[25] Yang R., Wu Y.N., Wang M., Sun Z.F., Zou J.H., Zhang Y.D. (2015). HDAC9 promotes glioblastoma growth via TAZ-mediated EGFR pathway activation. Oncotarget.

[26] Wang M., Liu Y., Zou J., Yang R., Xuan F., Wang Y. (2015). Transcriptional co-activator TAZ sustains proliferation and tumorigenicity of neuroblastoma by targeting CTGF and PDGF-beta. Oncotarget.

[27] Xiao H., Jiang N., Zhou B.Y., Liu Q., Du C.Y. (2015). TAZ regulates cell proliferation and epithelial–mesenchymal transition of human hepatocellular carcinoma. Cancer Sci..

[28] Zhao B., Ye X., Yu J., Li L., Li W., Li S. (2008). TEAD mediates YAP-dependent gene induction and growth control. Genes Dev..

[29] Sawada A., Kiyonari H., Ukita K., Nishioka N., Imuta Y., Sasaki H. (2008). Redundant roles of Tead1 and Tead2 in notochord development and the regulation of cell proliferation and survival. Mol. Cell. Biol..

[30] Plouffe S.W., Hong A.W., Guan K.L. (2015). Disease implications of the Hippo/YAP pathway. Trends Mol. Med..

[31] Sansores-Garcia L., Bossuyt W., Wada K.-I., Yonemura S., Tao C., Sasaki H. (2011). Modulating F-actin organization induces organ growth by affecting the Hippo pathway. Embo J..

[32] Dupont S., Morsut L., Aragona M., Enzo E., Giulitti S., Cordenonsi M. (2011). Role of YAP/TAZ in mechanotransduction. Nature.

[33] Bond M., Sala-Newby G., Wu Y., Newby A. (2006). Biphasic effect of p21Cip1 on vascular smooth muscle cell proliferation: role of PI3-kinase signalling and Skp2-mediated degradation. Cardiovasc. Res..

[34] Zhao B., Wei X., Li W., Udan R.S., Yang Q., Kim J. (2007). Inactivation of YAP oncoprotein by the Hippo pathway is involved in cell contact inhibition and tissue growth control. Genes Dev..

[35] Duggirala A., Newby A.C., Bond M. (2014). cAMP inhibits expression of the proangiogenic factor CCN1/CYR61 in vascular smooth muscle cells by inhibiting RhoA and actin-dependent regulation of MRTF. J. Mol. Cell. Cardiol..

[36] Lai D., Ho K.C., Hao Y., Yang X. (2011). Taxol resistance in breast cancer cells is mediated by the Hippo pathway component TAZ and its downstream transcriptional targets Cyr61 and CTGF. Cancer Res..

[37] Burdyga A., Conant A., Haynes L., Zhang J., Jalink K., Sutton R. (1833). cAMP inhibits migration, ruffling and paxillin accumulation in focal adhesions of pancreatic ductal adenocarcinoma cells: effects of PKA and EPAC. Biochim. Biophys. Acta Mol. Cell Res..

[38] Liu L., Xu X., Li J., Li X., Sheng W. (2011). Lentiviral-mediated shRNA silencing of PDE4D gene inhibits platelet-derived growth factor-induced proliferation and migration of rat aortic smooth muscle cells. Stroke Res. Treat..

[39] Koyama N., Morisaki N., Saito Y., Yoshida S. (1992). Regulatory effects of platelet-derived growth factor-AA homodimer on migration of vascular smooth muscle cells. J. Biol. Chem..

[40] Zhao B., Li L., Tumaneng K., Wang C.-Y., Guan K.-L. (2010). A coordinated phosphorylation by Lats and CK1 regulates YAP stability through SCF beta-TRCP. Genes Dev..

[41] Wu Y., Bond M., Sala-Newby G., Newby A. (2006). Altered S-phase kinase-associated protein-2 levels are a major mediator of cyclic nucleotide-induced inhibition of vascular smooth muscle cell proliferation. Circ. Res..

[42] Liu-Chittenden Y., Huang B., Shim J.S., Chen Q., Lee S.-J., Anders R.A. (2012). Genetic and pharmacological disruption of the TEAD–YAP complex suppresses the oncogenic activity of YAP. Genes Dev..

[43] Hernandez-Negrete I., Sala-Newby G.B., Perl A., Kunkel G.R., Newby A.C., Bond M. (2011). Adhesion-dependent Skp2 transcription requires selenocysteine tRNA gene transcription-activating factor (STAF). Biochem. J..

[44] Hewer R.C., Sala-Newby G.B., Wu Y.-J., Newby A.C., Bond M. (2011). PKA and Epac synergistically inhibit smooth muscle cell proliferation. J. Mol. Cell. Cardiol..

[45] Wang X., Hu G., Gao X., Wang Y., Zhang W., Harmon E.Y. (2012). The induction of yes-associated protein expression after arterial injury is crucial for smooth muscle phenotypic modulation and neointima formation. Arterioscler. Thromb. Vasc. Biol..

[46] Wang R., Xu Y.-J., Liu X.-S., Zeng D.-X., Xiang M. (2012). CCN2 promotes cigarette smoke-induced proliferation of rat pulmonary artery smooth muscle cells through upregulating cyclin D1 expression. J. Cell. Biochem..

[47] Shi Y., Fard A., Galeo A., Hutchinson H.G., Vermani P., Dodge G.R. (1994). Transcatheter delivery of c-myc antisense oligomers reduces neointimal formation in A porcine model of coronary-artery balloon injury. Circulation.

[48] Ota M., Sasaki H. (2008). Mammalian Tead proteins regulate cell proliferation and contact inhibition as transcriptional mediators of Hippo signaling. Development.

[49] Lamar J.M., Stern P., Liu H., Schindler J.W., Jiang Z.G., Hynes R.O. (2012). The Hippo pathway target, YAP, promotes metastasis through its TEAD-interaction domain. Proc. Natl. Acad. Sci. U.S.A..

[50] Jeong G.O., Shin S.H., Seo E.J., Kwon Y.W., Heo S.C., Kim K.H. (2013). TAZ mediates lysophosphatidic acid-induced migration and proliferation of epithelial ovarian cancer cells. Cell. Physiol. Biochem..

[51] Aragona M., Panciera T., Manfrin A., Giulitti S., Michielin F., Elvassore N. (2013). A mechanical checkpoint controls multicellular growth through YAP/TAZ regulation by actin-processing factors. Cell.

[52] Wang Y., Hu G., Liu F., Wang X., Wu M., Schwarz J.J. (2014). Deletion of yes-associated protein (YAP) specifically in cardiac and vascular smooth muscle cells reveals a crucial role for YAP in mouse cardiovascular development. Circ. Res..

[53] Xie C.Q., Guo Y.H., Zhu T.Q., Zhang J.F., Ma P.X., Chen Y.E. (2012). Yap1 protein regulates vascular smooth muscle cell phenotypic switch by interaction with myocardin. J. Biol. Chem..

[54] LAllemain G., Lavoie J., Rivard N., Baldin V., Pouyssegur J. (1997). Cyclin D1 expression is a major target of the cAMP-induced inhibition of cell cycle entry in fibroblasts. Oncogene.

[55] Haque I., De A., Majumder M., Mehta S., McGregor D., Banerjee S.K. (2012). The matricellular protein CCN1/Cyr61 is a critical regulator of sonic hedgehog in pancreatic carcinogenesis. J. Biol. Chem..

