# Supplementary material for: The Hippo pathway mediates inhibition of vascular smooth muscle cell proliferation by cAMP
Source: J Mol Cell Cardiol. 2016 Jan;90:1–10. doi: 10.1016/j.yjmcc.2015.11.024 (PMC4727789; doi:10.1016/j.yjmcc.2015.11.024)
Supplement: Supplementary file 1 — Supplementary material. [file mmc1.pdf]

SUPPLEMENTARY FIGURES

Supplement Figure 1

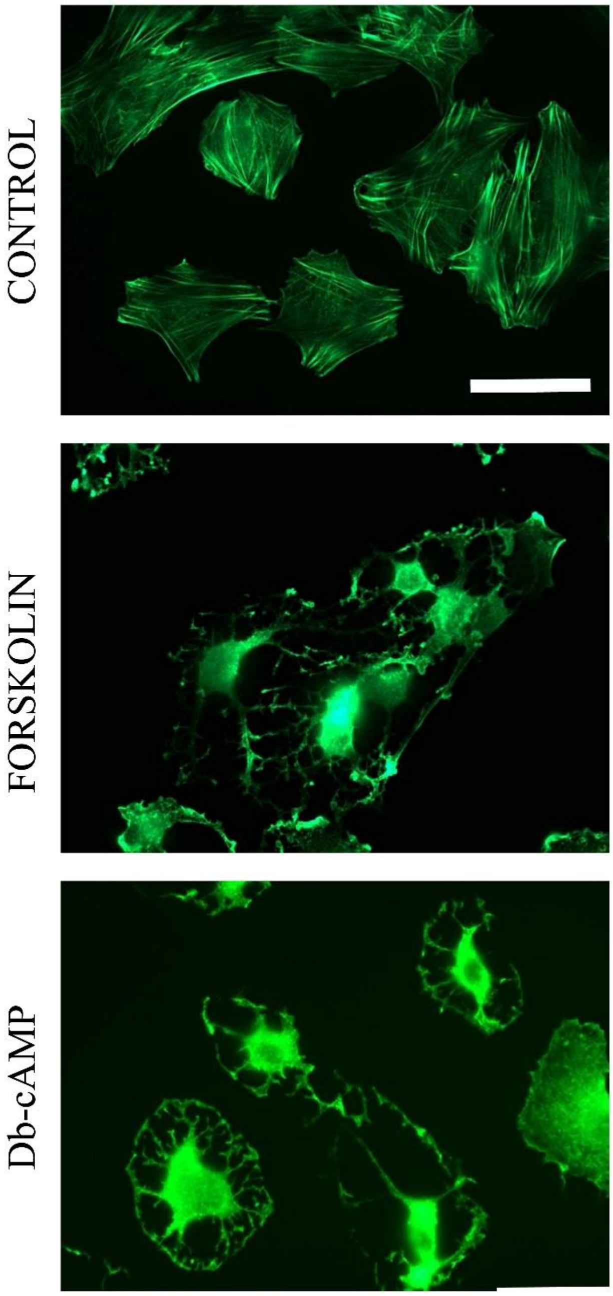

Supplement Figure 1: Phalloidin staining of actin stress fibres after forskolin or db-cAMP stimulation

Rat VSMC were sti,ulated with 25 µM forskolin of 500 µM db-cAMP for 60 minutes. Cells were fixed in paraformaldehyde and stained with Alexa-488 phalloidin. Bar indicates 50 µm.

Supplement Figure 2

Supplement Figure 2

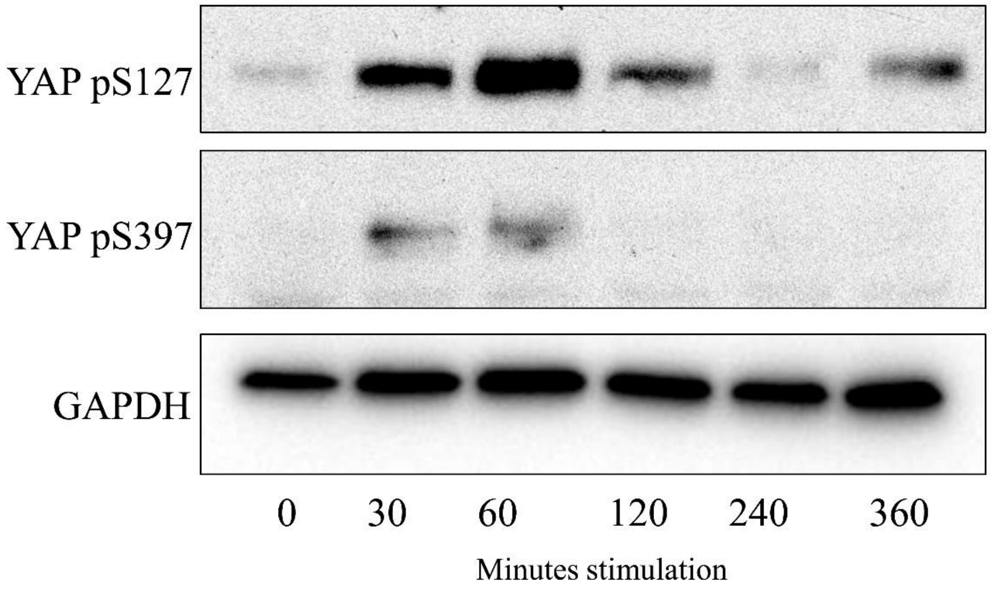

Supplement Figure 2: Forskolin stimulation induces YAP phosphorylation in Human VSMC

Human aortic VSMC were stimulated with 25 μM forskolin for the indicated times and YAP phospho-S127 and phospho-S397 phosphorylation quantified by Western blotting.

Supplement Figure 3

Supplement Figure 3

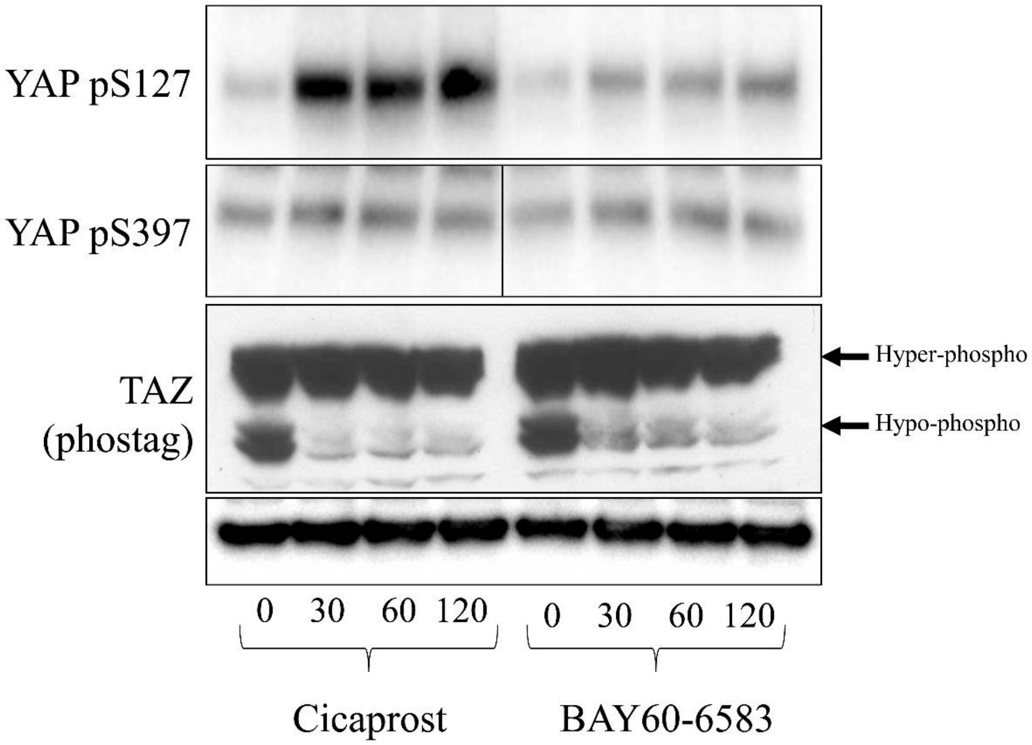

Supplement Figure 3: The GPCR-agonists Cicaprost and BAY60-6583 stimulate YAP and TAZ phosphorylation

Rat aortic VSMC were rendered quiescent by serum deprivation for 4 hours followed by stimulation with 1 µM Cicaprost or 1 µg/ml BAY60-6583 for the indicated times and YAP phospho-S127, phospho-S397 and pan-TAZ phosphorylation quantified by Western blotting.

Supplement Figure 4

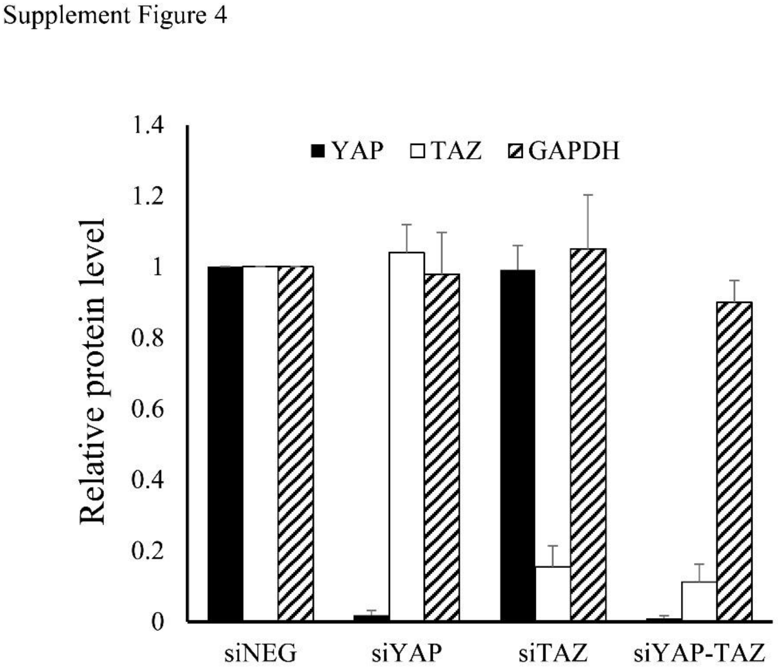

Supplement Figure 4: Specific silencing of YAP and TAZ using siRNA

Rat aortic VSMC (1x10^6 cells) were transfected with 50 pmoles of YAP siRNA plus 50 pmoles TAZ siRNA. 24 hours later total RNA was analysed for mRNA levels of indicated genes using qRT-PCR.

Supplement Figure 5

Supplement Figure 5

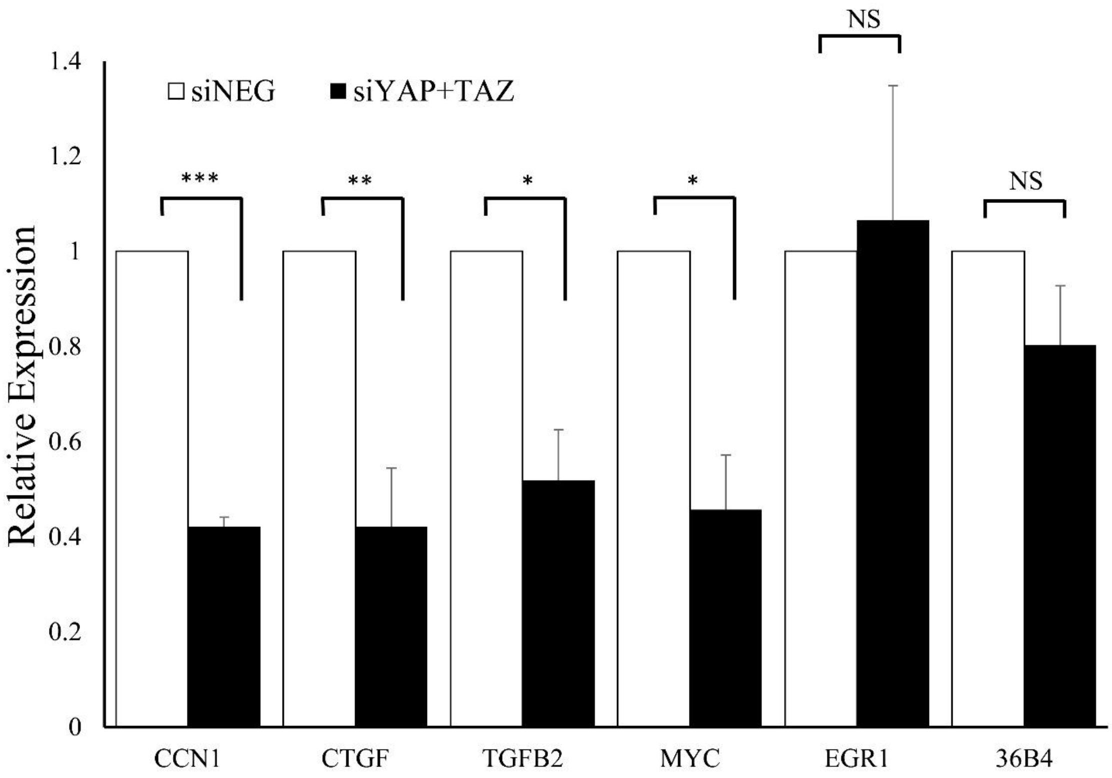

Supplement Figure 5: YAP/TAZ-dependence of selected proliferation genes in VSMC

Rat aortic VSMC ($1x10^6$ cells) were transfected with 50 pmoles of YAP siRNA plus 50 pmoles TAZ siRNA. 24 hours later total RNA was analysed for mRNA levels of indicated genes using qRT-PCR.

Supplement Figure 6

Supplement Figure 6

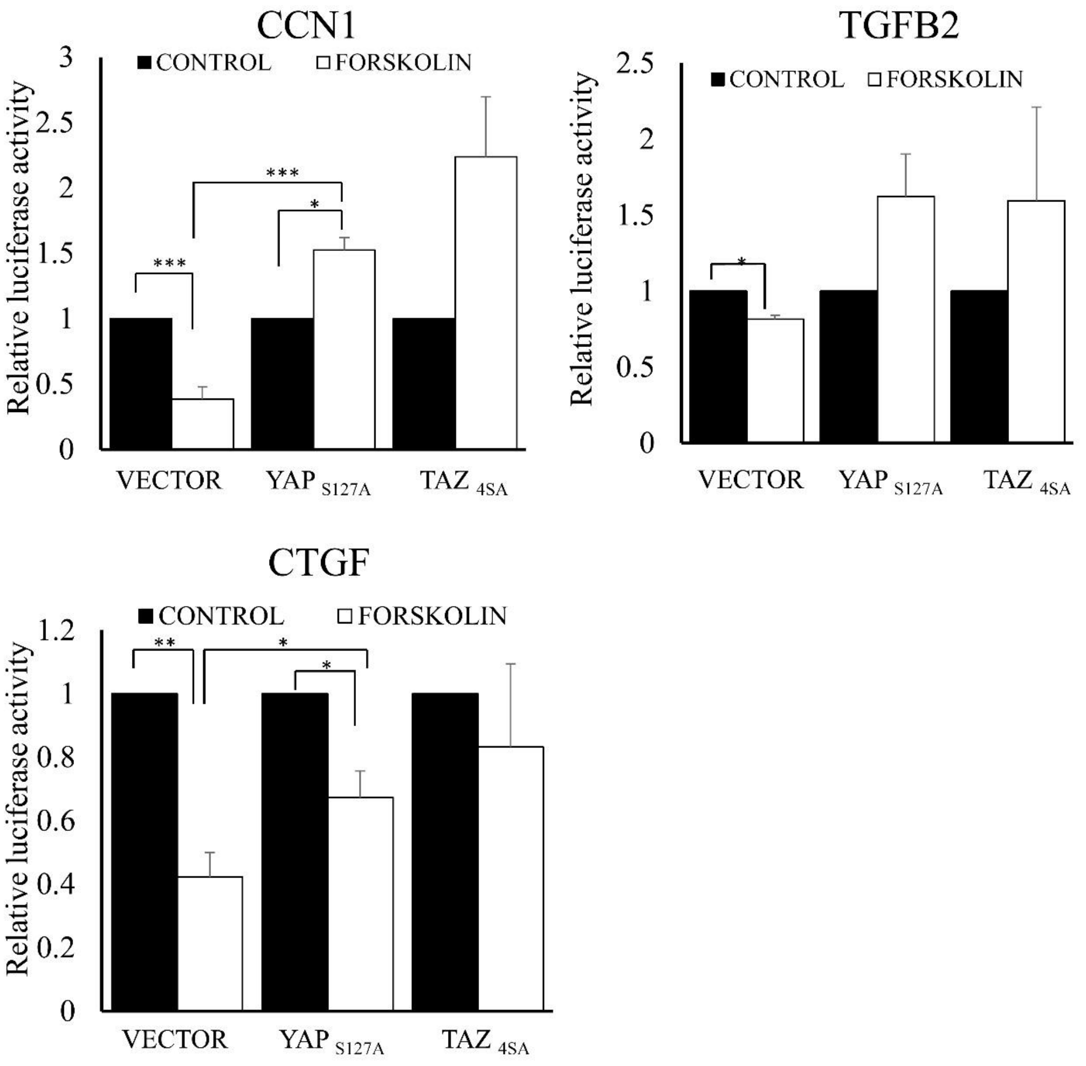

Supplement Figure 6: Constitutively-active YAP and TAZ rescue CCN1, CTGF and TGFB2 promoter activity after forskolin stimulation.

Rat aortic VSMC were transfected with either CCN1-LUC, CTGF-LUC or TGFB2-LUC promoter reporter gene plasmids. Transfected cells were stimulated for 8 hours with 25 μM forskolin and luciferase activity quantified.

Supplement Figure 7

Supplement Figure 7

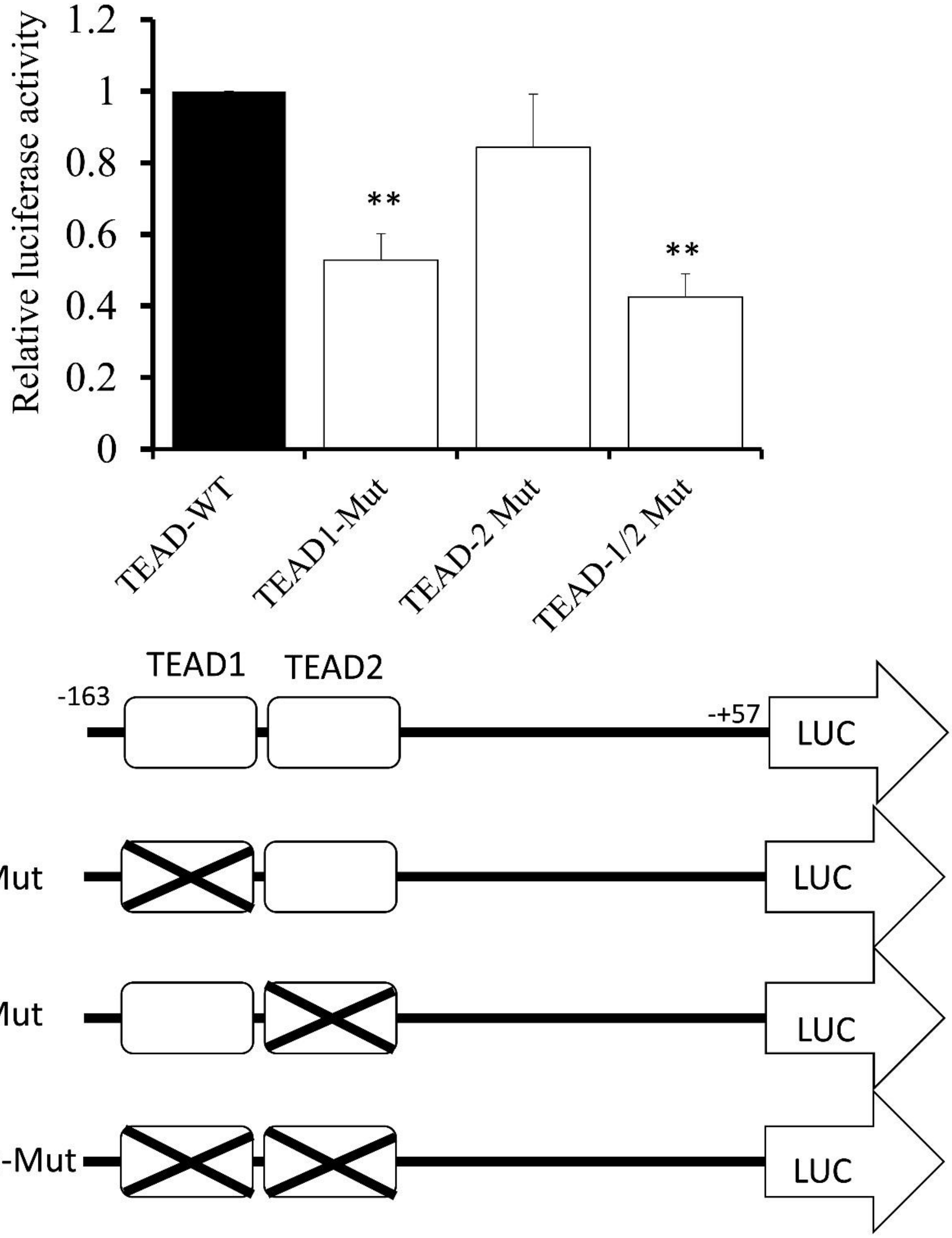

Supplement Figure 7: Mutational analysis of the CCN1 promoter proximal TEAD-elements
Rat VSMC were transfected with the indicated *CCN1-LUC* reporter vectors containing either wild-type of mutated TEAD elements. Luciferase activity was quantified 24 hours post infection.

Supplement figure 8

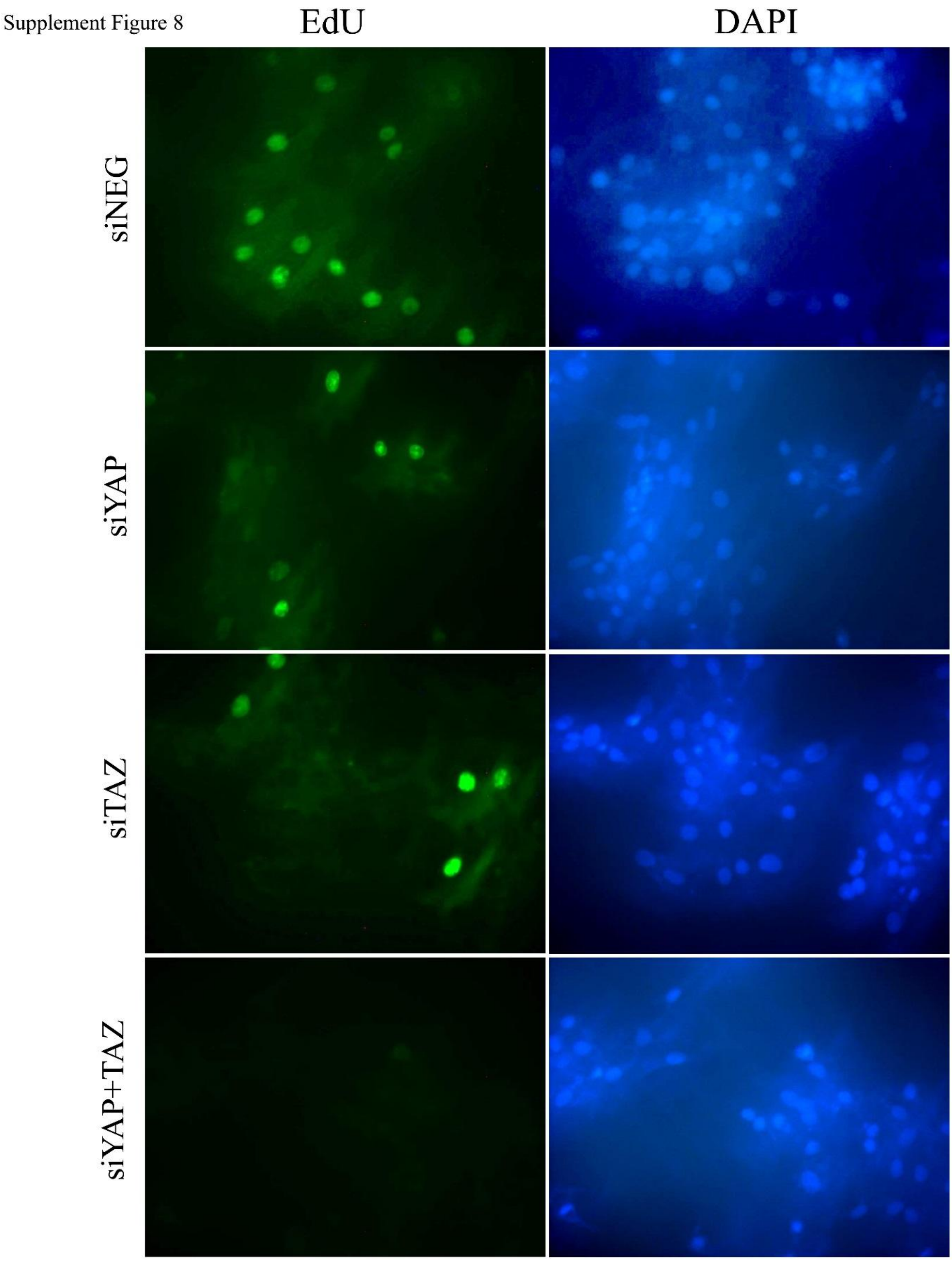

Supplement Figure 8: **Representative EdU-histograms of cells transfected with YAP and or TAZ siRNA**

Cells were treated as described in Figure 6C. Representative EdU-histograms of cells transfected with YAP and or TAZ siRNA are shown.

Supplement figure 9

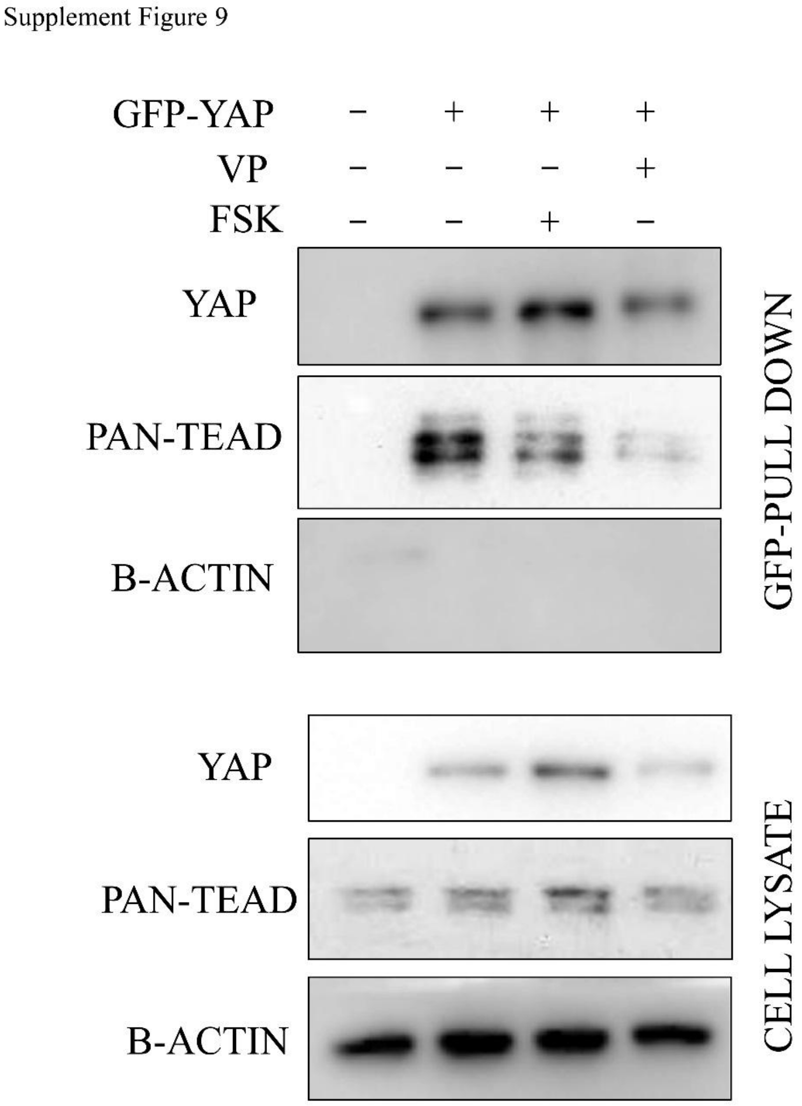

Supplement figure 9: Specific silencing of YAP and TAZ using siRNA
Rat aortic VSMC were transfected with GFP-YAP plasmid. 24 hours later cells were stimulated with 25 uM forskolin or 10 µVerteporfin (VP) for 2 hours. GFP-YAP and co-interacting proteins were affinity purified using GFP-Trap. Isolated proteins were quantified by Western blotting.

Supplement figure 10

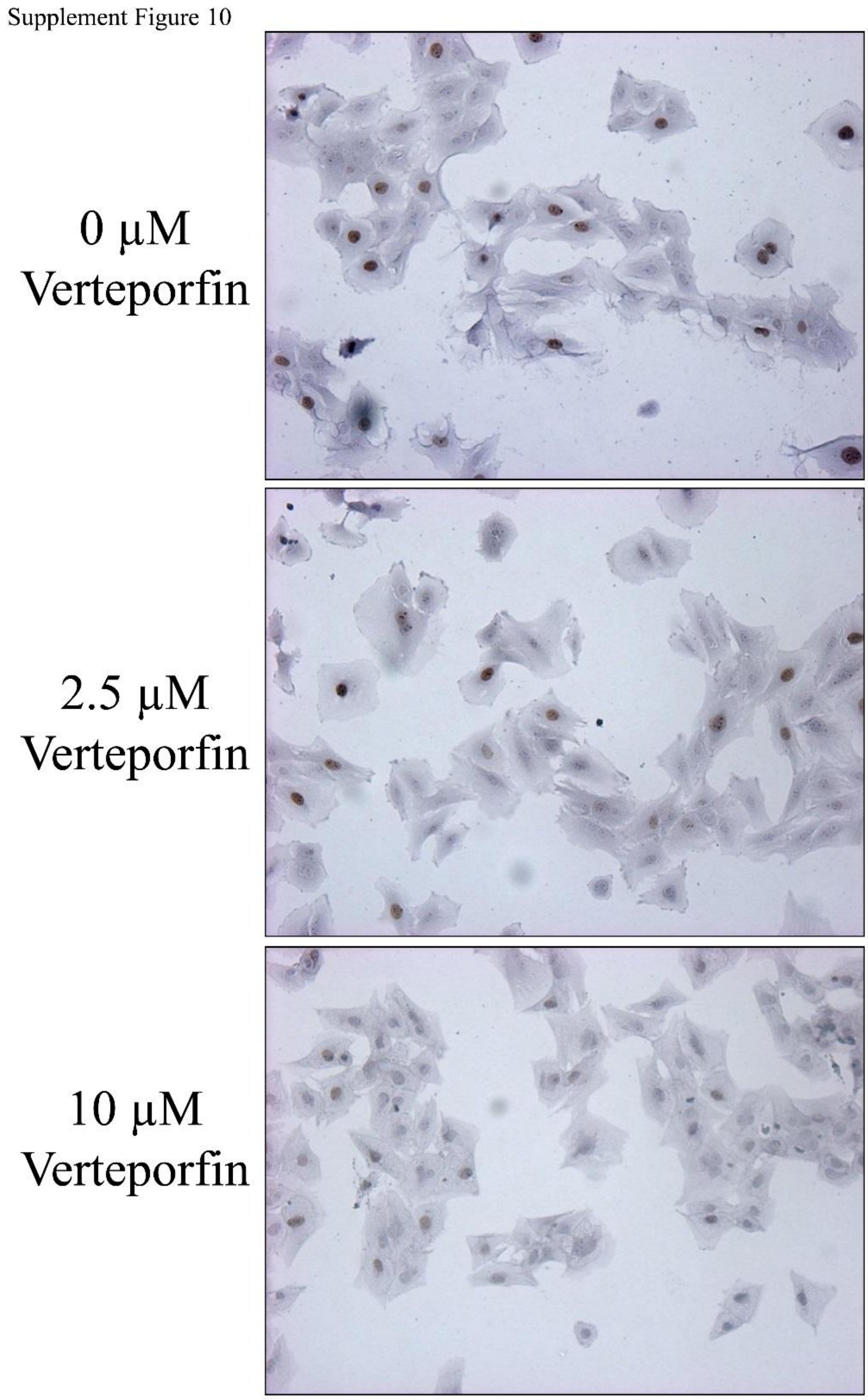

Supplement Figure 10: Representative BrdU-histograms of cells treated with Verteporfin
Cells were treated as described in Figure 7D. Representative BrdU-histograms of cells treated with indicated doses of Verteporfin are shown.

Supplement figure 11

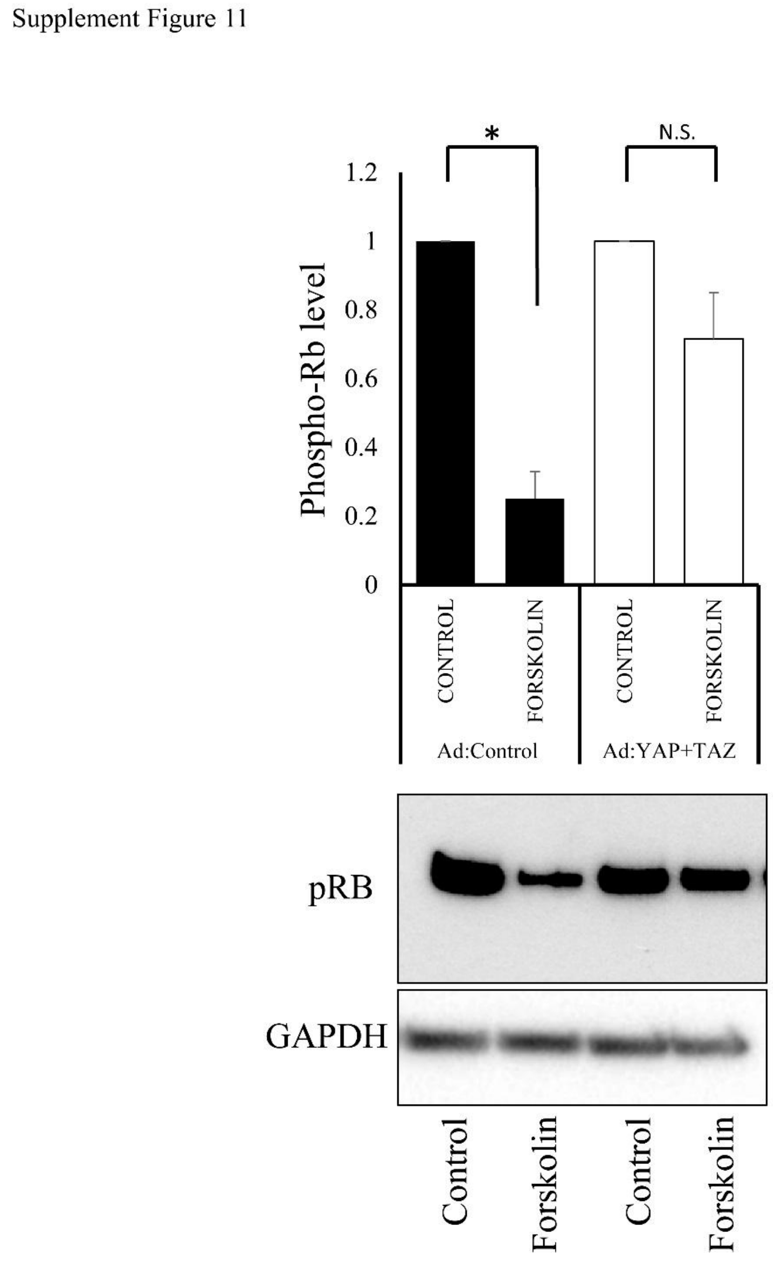

Supplement Figure 11: Constitutively active YAP and TAZ reverses the anti-mitogenic effect of forskolin in VSMC

VSMC were infected with 6x10^7 pfu/ml of control adenovirus or 3x10^7 pfu/ml of each adenovirus expressing constitutively active YAP$_{S127A}$ and TAZ$_{4SA}$. Cells were stimulated with 25 µM forskolin for 18 hours. Total cell lysates were analysed for phospho-Rb levels by Western blotting and densitometry.

Supplement figure 12

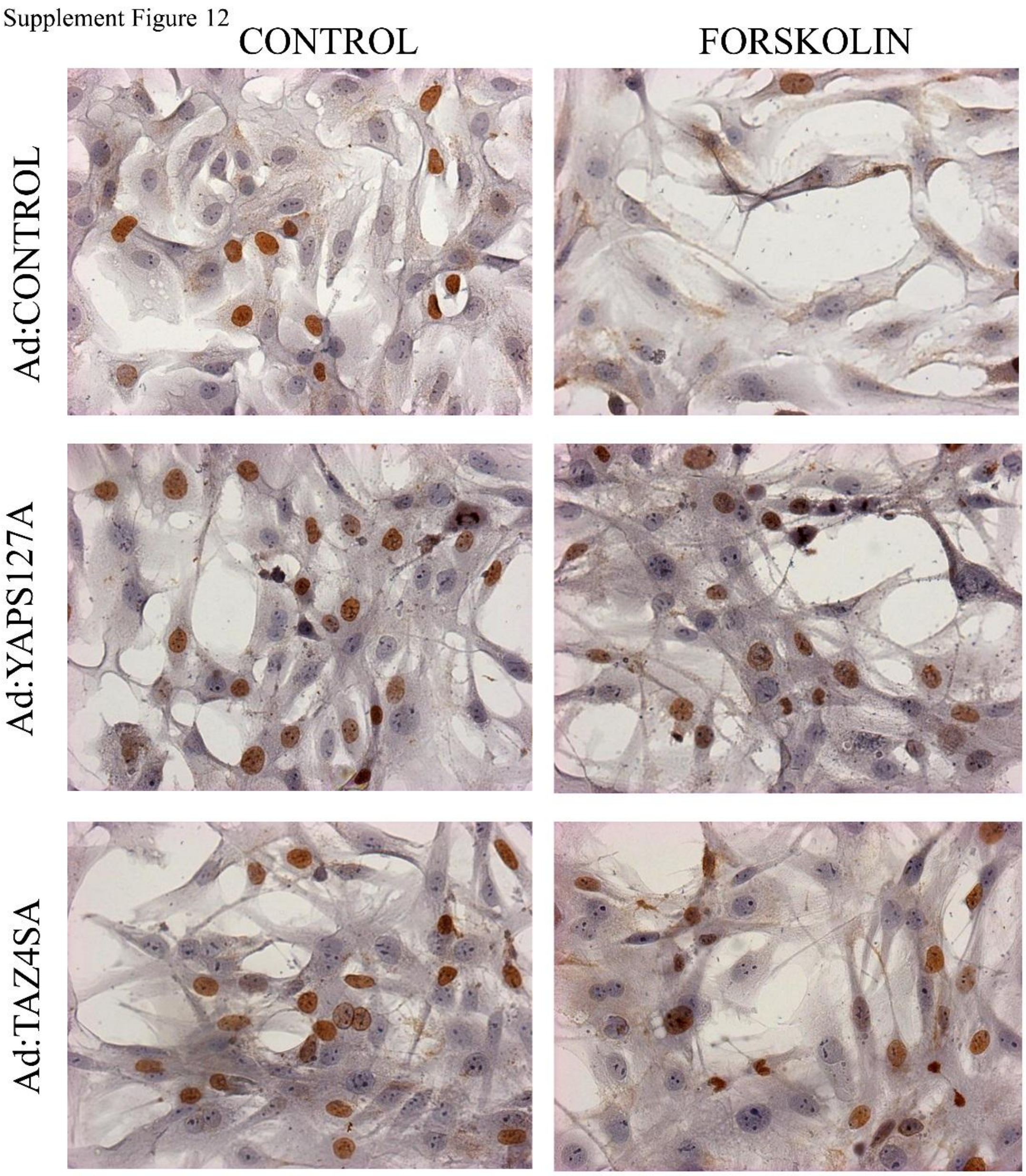

Supplement Figure 12: **Representative BrDU histograms of cells expressing YAP_{S127A} or TAZ_{4SA} after forskolin stimulation**

Cells were treated as described in figure 8B. Representative BrDU histograms are shown.

Supplement Table 1

Primer description	Seqcuence (5'-3')
Rat CCN1 forward	GGAACTGGCATCTCCACACGAGTT
Rat CCN1 reverse	TTGTCCACAAGGACGCACTTCACA
Rat CTGF forward	GGAAATGCTGTGAGGAGTGGGTGT
Rat CTGFreverse	TGTCTTCCAGTCGGTAGGCAGCTA
Rat cMYC forward	GAGGAGAAACGAGCTGAAGCGTAG
Rat cMYC reverse	TTCTCGCCGTTTCCTCAGTAAGTC
rat TGFB2 forward	TCCATACAGTCCCAGGTGCTCTGT
rat TGFB2 reverse	GACCCTGAACTCTGCCTTCACCAG

Supplement Table 1: qPCR primer sequences.
